# Combining Machine Learning and Metabolomics to Identify Weight Gain Biomarkers

**DOI:** 10.3389/fbioe.2020.00006

**Published:** 2020-01-24

**Authors:** Flávia Luísa Dias-Audibert, Luiz Claudio Navarro, Diogo Noin de Oliveira, Jeany Delafiori, Carlos Fernando Odir Rodrigues Melo, Tatiane Melina Guerreiro, Flávia Troncon Rosa, Diego Lima Petenuci, Maria Angelica Ehara Watanabe, Licio Augusto Velloso, Anderson Rezende Rocha, Rodrigo Ramos Catharino

**Affiliations:** ^1^Innovare Biomarkers Laboratory, School of Pharmaceutical Sciences, University of Campinas, Campinas, Brazil; ^2^RECOD Laboratory, Institute of Computing (IC), University of Campinas, Campinas, Brazil; ^3^Centro Universitário Filadélfia, Londrina, Brazil; ^4^Laboratory of Studies and Applications of DNA Polymorphisms, Biological Sciences Center, Londrina State University, Londrina, Brazil; ^5^Department of Internal Medicine, School of Medical Sciences, University of Campinas, Campinas, Brazil

**Keywords:** obesity, machine learning, random forest, metabolomics, biomarkers

## Abstract

Weight gain is a metabolic disorder that often culminates in the development of obesity and other comorbidities such as diabetes. Obesity is characterized by the development of a chronic, subclinical systemic inflammation, and is regarded as a remarkably important factor that contributes to the development of such comorbidities. Therefore, laboratory methods that allow the identification of subjects at higher risk for severe weight-associated morbidity are of utter importance, considering the health, and safety of populations. This contribution analyzed the plasma of 180 Brazilian individuals, equally divided into a eutrophic control group and case group, to assess the presence of biomarkers related to weight gain, aiming at characterizing the phenotype of this population. Samples were analyzed by mass spectrometry and most discriminant features were determined by a machine learning approach using Random Forest algorithm. Five biomarkers related to the pathogenesis and chronicity of inflammation in weight gain were identified. Two metabolites of arachidonic acid were upregulated in the case group, indicating the presence of inflammation, as well as two other molecules related to dysfunctions in the cycle of nitric oxide (NO) and increase in superoxide production. Finally, a fifth case group marker observed in this study may indicate the trigger for diabetes in overweight and obesity individuals. The use of mass spectrometry combined with machine learning analyses to prospect and characterize biomarkers associated with weight gain will pave the way for elucidating potential therapeutic and prognostic targets.

## Introduction

Weight gain has become a worldwide epidemic, leading to a population of obese individuals of more than 13% of the world population in 2016. According to the World Health Organization, obesity has almost tripled since 1975 and is now linked to a larger number of deaths than underweight alone (World Health Organization, 2018). Obesity is characterized by weight gain, excess body fat and complications in several tissues, and organs. The condition is associated with type 2 diabetes, hypertension, dyslipidemia and cardiovascular events (Libert et al., 2018). The presence of three or more of these disorders (including elevated fasting glucose and abdominal obesity) define Metabolic Syndrome (MetS), a multifactorial disease that increase substantially the mortality risk (Zhong et al., 2017; Lent-Schochet et al., 2019). According to a MetS prevalence study, 34% of Americans meet the diagnose standards, placing MetS as an important public health issue (Aguilar et al., 2015). Considering the wide variety of possible complications and the different states of metabolic disorders in MetS, it is important to have a better understanding of the metabolic environment of these conditions. In addition, the different profiles found in overweight individuals makes the search for biomarkers - essential for future definitions of diagnosis, prognosis, and treatment (Zhong et al., 2017; Libert et al., 2018).

Weight gain results from a chronic state of positive energy balance due to increased caloric intake and reduced energy expenditure, and may be influenced by a number of genetic and environmental factors (Velloso et al., 2015). Adipose tissue expansion and hypertrophied adipocytes tend to activate intra- and extracellular stress responses, promoting the development of a tissue-specific and systemic proinflammatory state (Sartipy and Loskutoff, 2003; Cooke and Naaz, 2004; Guilherme et al., 2008). This state of metabolic disturbance, activation of immune system and increase in inflammation markers in overweight and obesity is described as a low-grade chronic inflammation, and recent studies have demonstrated some of the pathways associated with this condition. Human and murine research has evinced the relationship between increased nutrient intake, weight gain and inflammatory responses (Gregor and Hotamisligil, 2011; Andersen et al., 2016). Despite that, it is still necessary to unravel the many metabolic and molecular pathways involved in the development of obesity. In this sense, studies in the field of “omics” have provided advance in the physiological and pathological alterations in living organisms. Considering that metabolomics is the study of all metabolites within an organism, including precursors, intermediates and end products, and that mass spectrometry produces a large amount of data for each sample, machine learning techniques provide tools to recognize patterns in this large data environment. In this way, the use of mass spectrometry with machine learning builds an experimental metabolomics platform to develop diagnostics and prevention tools (Kononenko, 2001; Jordan and Mitchell, 2015).

Within this context, this study aims at elucidating molecules that may be involved in the development of obesity in a cohort of overweight and obese individuals and propose the relationship of such species with chronical inflammation. Finally, we understand this study as the basis for potential developments and applications of targeted therapeutics and clinically-relevant prognostic markers.

## Materials and Methods

### Ethics Statement

This study was conducted according to the principles expressed in the Declaration of Helsinki and approved by the Research Ethics Committee of the State University of Londrina, Paraná, Brazil (No. 2.426.419—No. CAAE: 79277817.8.0000.5231). A written informed consent was obtained from all patients. Samples were obtained from the Health Education Center of Filadelfia University Center—UNIFIL, Londrina, Paraná, Brazil and State University of Londrina, Paraná, Brazil.

### Research Participants and Specimen Collection

In total, 180 patients were included in this study, separated into two groups: Case, formed by a combination of obese and overweight individuals, and Control, composed of eutrophic individuals. A questionnaire was used to collect data such as age, gender, and personal and family history of chronic non-communicable diseases. A nutritional assessment was performed including weight and height. The collected specimens from all participants of the present study consisted of blood (plasma) samples.

Body Mass Index (BMI) was calculated as weight in kilograms divided by height in meters squared. Overweight and Obese (case group) classifications were defined based on the respective BMI standard cut points that have been recommended by the World Health Organization (WHO) (World Health Organization, 1995): BMIs of ≥25 and <30 kg/m^2^ for Overweight; BMIs of ≥30 and <34.9 kg/m^2^ for class I obesity (*n* = 29); BMIs of ≥35 and <39.9 kg/m^2^ for class II obesity (*n* = 17); BMIs ≥ 40 kg/m^2^ for class III obesity (*n* = 13).

For the control group, eutrophic individuals that presented a BMI between ≥18 and <24.9 kg/m^2^ were recruited. In order to exclude from the group any individuals with normal BMI, but with fat percentage above normal, an estimation of body fat composition was performed using a bivalent (foot to foot) bioimpedance analysis (Electronic Scale BC533, Tanita, Tokyo, Japan and Electronic Scale W905, Wiso, São José, Brazil). The percentage of fat within the average for age and gender were determined according to Jackson and Pollock (1978) and Jackson et al. (1980) protocols.

### MALDI-MS Analysis

For sample preparation, 5 μL of plasma and 5 μL of methanol were mixed in a plastic tube. This solution was diluted in 90 μL of a MALDI matrix α-cyano-4hydroxycinnamic acid (Sigma-Aldrich, St. Louis, MO) solution at 10 mg/mL, prepared in 1:1 acetonitrile/methanol. Then, 1 μL of the obtained solution was spotted on a MALDI 96-well plate and air-dried. Each sample was analyzed in quintuplicates. Spectra were acquired using a MALDI LTQ-XL (Thermo Scientific, San Jose, CA) at the mass range of 250–2,000 m/z, in the positive ion mode.

### Machine Learning Method

Machine learning has been intensively used in MS imaging (Hanselmann et al., 2009; Rappez et al., 2019), and is becoming a key methodology in untargeted metabolomics (Li et al., 2019). The strategy applied in this article is an extension of the ML-based platform used by our group for screening ZIKV molecules in blood serum (Melo et al., 2018). The method ranks variables using the feature importance measure provided by Random Forest trained trees (Breiman et al., 1984; Breiman, 2001; Gregor and Hotamisligil, 2011). It also evaluates the ranking results when discarding least important variables iteratively. Using f1-score (first harmonic average on recall and precision) as the cost function (performance measure), it is possible to determine which features on the mass spectra are most discriminant to predict positive patients based on their observations (Melo et al., 2018). A positive patient is defined as any given individual with the condition of interest herein, i.e., weight gain. At the end of the process, the method provides a Random Forest classifier, called diagnosis classifier, that can predict weight gain markers based on the blood serum mass spectrum, as well as which features are the most important on that prediction, determining the starting point for the metabolomics research.

Starting with data preparation, all vectors were normalized dividing intensity values by the maximum one in each vector. Then, we randomly split the patient samples into two partitions randomly selected, the ***P***_***test***_ partition, corresponding to 20% of the all patients, i.e., data from 18 controls and 18 patients with weight gain. This partition was kept untouched until the end of the process, when it was used to measure the classification accuracy and precision of the classifier, as well as the potential biomarkers identified by the process. The remaining partition ***P***_***fit***_ consisted of 80% of patients' data points (82 controls and 82 patients with weight gain) and were used to determine the hyperparameters of the machine-learning algorithm, aiming at ultimately training the classifier. For hyperparameters and determination of the most discriminant features, the ***P***_***fit***_ partition was randomly shuffled into 10 experiments datasets with 80% for training (65 cases and 65 controls) and 20% validation (17 controls and 17 patients with weight gain) in each experiment. It is important to state that vectors (mass spectra measurements) of the same patient were always in the same partition, so as not to contaminate the training and tests results.

A group of linearly dependent features did not carry more information than a single representative of the group; therefore, in order to enable the following process and improve resource consumption and time in the next steps, linearly dependent features were eliminated using the Gauss-Jordan elimination algorithm, keeping only features with maximum median across all vectors to represent each group. Thus, the following machine learning steps on most discriminant and putative biomarker features determination rely only on linearly independent features. In the search for molecules based on the determined *m/z*, dependent features are also used to help on the match of ions for the molecule represented by linearly independent representatives. After the elimination, 713 ions remained represented in the feature vectors from the original 1,752 *m/z* values.

**Figure 1:**
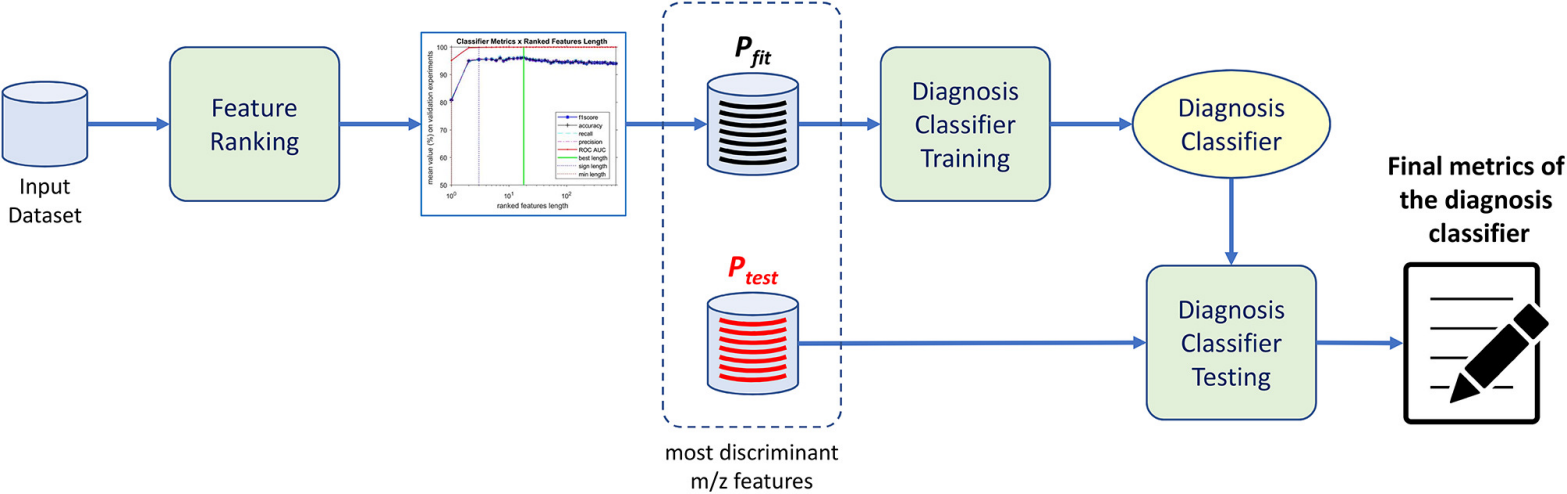
Diagnosis classifier training and testing procedure. Upon receiving data from different patients, we train the proposed method and rank the most important features through an analysis of feature distribution. Thereafter a classifier is trained with the selected featured of interest yielding the diagnosis classifier ready to evaluate different patients data.

**Figure 2:**
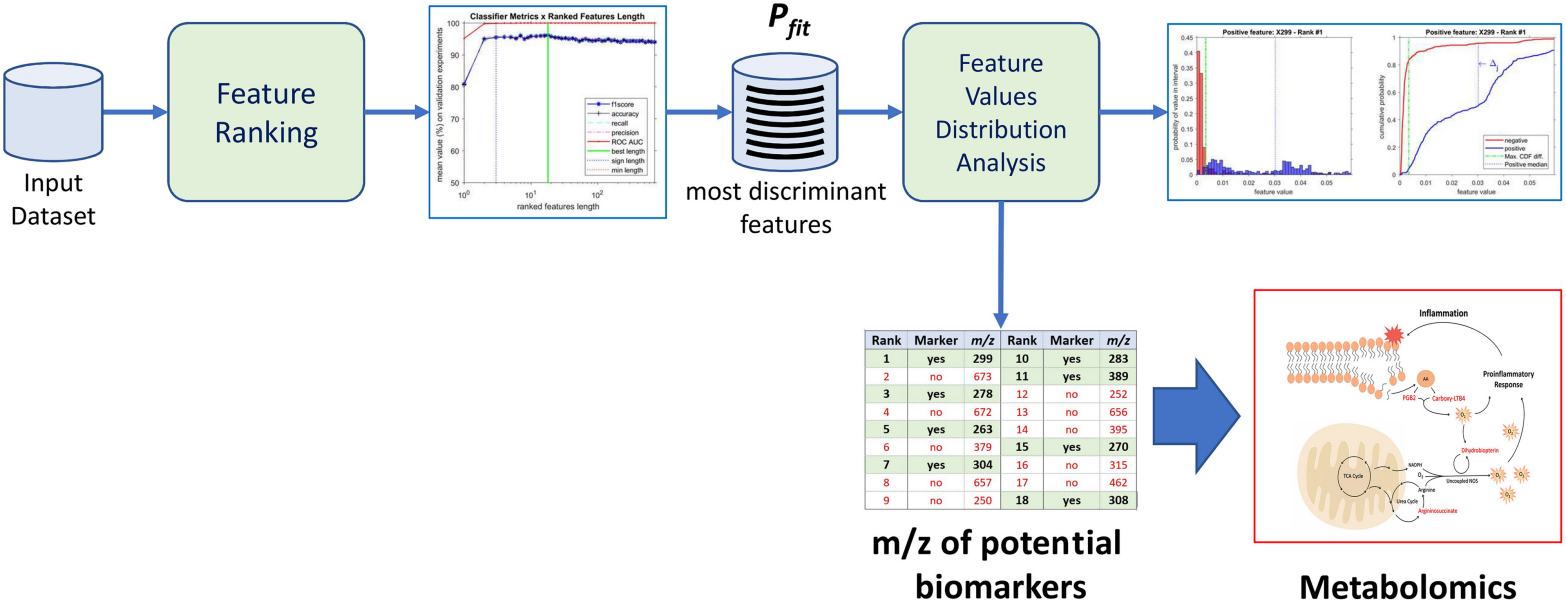
Identification of potential biomarkers through the proposed machine-learning process using the most discriminant features as a proxy.

The purpose of the machine-learning process here devised is 2-fold: first, to generate a diagnosis classifier, which will be used for screening patients to the studied condition (Figure 1); second, to determine potential biomarkers, which will follow to the metabolomics analysis stage, reducing the number of molecules to be analyzed (Figure 2). In the proposed machine learning approach, Random Forest algorithm plays an important role. In addition to be a good choice for a classifier, as shown by Fernández-Delgado et al. (2014), it also provides the variables' importance, which allows them to be ranked based on their influence on the classification results.

Statistical metrics defined in Table 1 are used to evaluate the classifiers' performance on the experiments and final tests. *Tp* denotes true-positive examples, which are cases that actual positives are classified correctly as positives. *Tn* stands for true-negative cases, which are examples that actual negatives are classified correctly as negatives. Conversely, *Fp* denotes false positive cases (those that are actual negatives and were wrongly classified as positives). Finally, *Fn* represents the false negative examples (those examples that are actual positives, but were incorrectly classified as negatives).

**Table 1:** Statistical metrics definition to evaluate classification results.

**Metric**	**Sensitivity (Stv) or Tpr**	**Specificity (Spc)**	**Precision (Prc)**	**F1-score (F1s)**	**Accuracy (Acc)**
Formula	*Tp*/*Tp*+*Fn*	*Tn*/*Tn*+*Fp*	*Tp*/*Tp*+*Fp*)	2·*Prc*·Stv /*Prc*+ Stv	(*Rcl*+*Spc*)/2

Before starting to determine the most discriminant features, we selected the appropriate hyperparameter *number of trees* in the Random Forest method through a grid-search procedure, seeking to maximize the accuracy and f1-score as function of vector length and number of trees. The feature ranking step measures classification results averaging statistical metrics of the trained classifiers' validation (through the 10 repetitions previously described). In each interaction, the metrics on Table 1 were determined, and features were ranked according to their feature importance. Vector length was reduced by a factor of 0.9 in each interaction, discarding the less significant features until it achieved length 1.

Marker features represents the ions that are potential biomarkers to be further analyzed in metabolomics research. From the 18 most discriminant features, eight were identified as markers by the definition (see equation below) that a marker feature has the difference between the cumulative distribution function (CDF) of the feature values for the negative class and the CDF of positive class vectors, at the median value of positive class, exceeding the threshold **β**, that was defined as 0.4 (40%) for the analysis herein.

$$\begin{array}{l} \;F_{j}\;is\;a\;marker\;feature,\;\;if:\;\\ \Delta_{j}=\;\left(1-P\left(m_{j}\right)\right)-\left(1-\;Q\left(m_{j}\right)\right)=\;Q\left(m_{j}\right)-P\left(m_{j}\right)>\beta \; \end{array}$$

and

$$\begin{array}{l} Q\left(F_{j}\right)\geq P\left(F_{j}\right)\;|\;\forall \;F_{j}>m_{j}\; \end{array}$$

where

*y*_*j*_ is a *F*_*j*_ value for a positive patient;

*m*_*j*_ is the median of *y*_*j*_, i.e., median of *F*_*j*_ values of positive patients;

${\bar{y}}_{j}$ is a *F*_*j*_ value for a negative patient;

*p*(*y*_*j*_) is the probability distribution function of positive patients, and $q\left({\bar{y}}_{j}\right)$ the probability distribution function of negative patients;

*P*(*y*_*j*_) is the cumulative distribution function (CDF) of y values, and $Q\left({\bar{y}}_{j}\right)$ is the CDF of ${\bar{y}}_{j}$;

0 < β < 0.5 CDF is the threshold of the difference over median of the feature j for the positive patients (e.g., β = 40%).

To evaluate how the most discriminant features (18 variables) perform in different classifiers, we submitted the same partitions used to train and validate the Random Forest classifier to a SVM classifier with two different optimization algorithms: (SMO) Sequential Minimal Optimization (Fan et al., 2005), and (ISDA) Iterative Single Data Algorithm (Kecman et al., 2005), and to a single decision tree classifier using (GDI) Gini Diversity Index (Breiman et al., 1984) as the split criterion (same criterium used by the Random Forest).

The software platform used for machine learning processing was the MATLAB 2018b version 9.5.0.1067069 (R2018b) February 28, 2019 Update 4, especially the bagged ensemble decision tree algorithm and related functions available in the package Statistics and Machine Learning Toolbox. All experiments ran on a Samsung Notebook NP500R5H with Intel® Core™ i7-5500U CPU @ 2.40 GHz, RAM 8 GB on Windows 10 v1903.

The datasets and codes used to perform these experiments are available in public repository through the link http://dx.doi.org/10.21227/k446-fp12 (Dias-Audibert et al., 2019).

### HRMS Analyses

In order to determine the exact masses of the markers discriminated by machine learning, case group samples were analyzed in an ESI-LTQ-XL Orbitrap Discovery (Thermo Scientific, Bremen, Germany). For sample preparation, following an adapted protocol by Melo et al. (2017), 20 μL of plasma were diluted in 200 μL of tetrahydrofuran and homogenized under vortex for 30 s; the volume was completed to 1 mL with methanol, with further homogenization, and then centrifuged for 5 min under 3,600 rpm. Hundred microliter of the supernatant was diluted in 400 μL of methanol. Formic acid was added to 0.1% in the final solution. Samples were directly infused into an ESI-LTQ-XL Orbitrap Discovery (Thermo Scientific, Bremen, Germany), and data were acquired according to the following parameters: flow rate at 10 μL.min^−1^, capillary temperature at 280°C, spray current at 5 kV, and sheath gas at five arbitrary units, in the mass range of 200–2,000 *m/z*.

### Structure Elucidation

Human Metabolome database version 3.6 (www.hmdb.ca), LipidMaps (www.lipidmaps.org), and METLIN (Scripps Center for Metabolomics, La Jolla, CA) were consulted to elect the most suitable marker obtained from HRMS and mass accuracy; only molecules with a mass shift lower than 2 ppm were considered. Aiming to confirm the proposed molecules, *Tandem* mass spectrometry (MS/MS) data were acquired and structure proposals were carried out with the assistance of METLIN MS/MS databases and fragmentation calculations/ modeling predicted by Mass Frontier software (v. 6.0, Thermo Scientific, San Jose, CA).

## Results

Anthropometric data of the study groups are shown as means in Table 2. Regarding non-communicable diseases, the control group had no comorbidities, whereas in the 90 patients from case group, 37.5% had hypertension and 17.5% had diabetes. Mass spectral data obtained from 180 patient samples in MALDI-MS measurements fed the machine learning process with 893 (about 5 per patient) feature vectors of length 1,752 (*m/z* values as features).

**Table 2:** Anthropometric data of subjects.

**Metric**	**Group**	**All**		**Men**		**Women**	
		**Mean**	**RSD**	**Mean**	**RSD**	**Mean**	**RSD**
No. patients	Control	90		31		59	
	Case	90		18		72	
Age (y)	Control	35.1	12.5	34.5	13.2	35.4	12.2
	Case	39.0	15.1	44.3	17.2	37.7	14.4
Weigh (kg)	Control	64.0	11.1	75.3	8.2	57.8	6.7
	Case	88.6	18.9	103.5	22.5	84.8	16.0
BMI (kg/m^2^)	Control	22.3	2.8	24.7	2.3	21.0	2.0
	Case	33.3	6.5	34.4	9.3	33.0	5.7
Body fat percentage (%)	Case	40.2	7.6	33.7	5.9%	41.7	7.2

### Ranked Results of Ml Analyses

**Figure 3:**
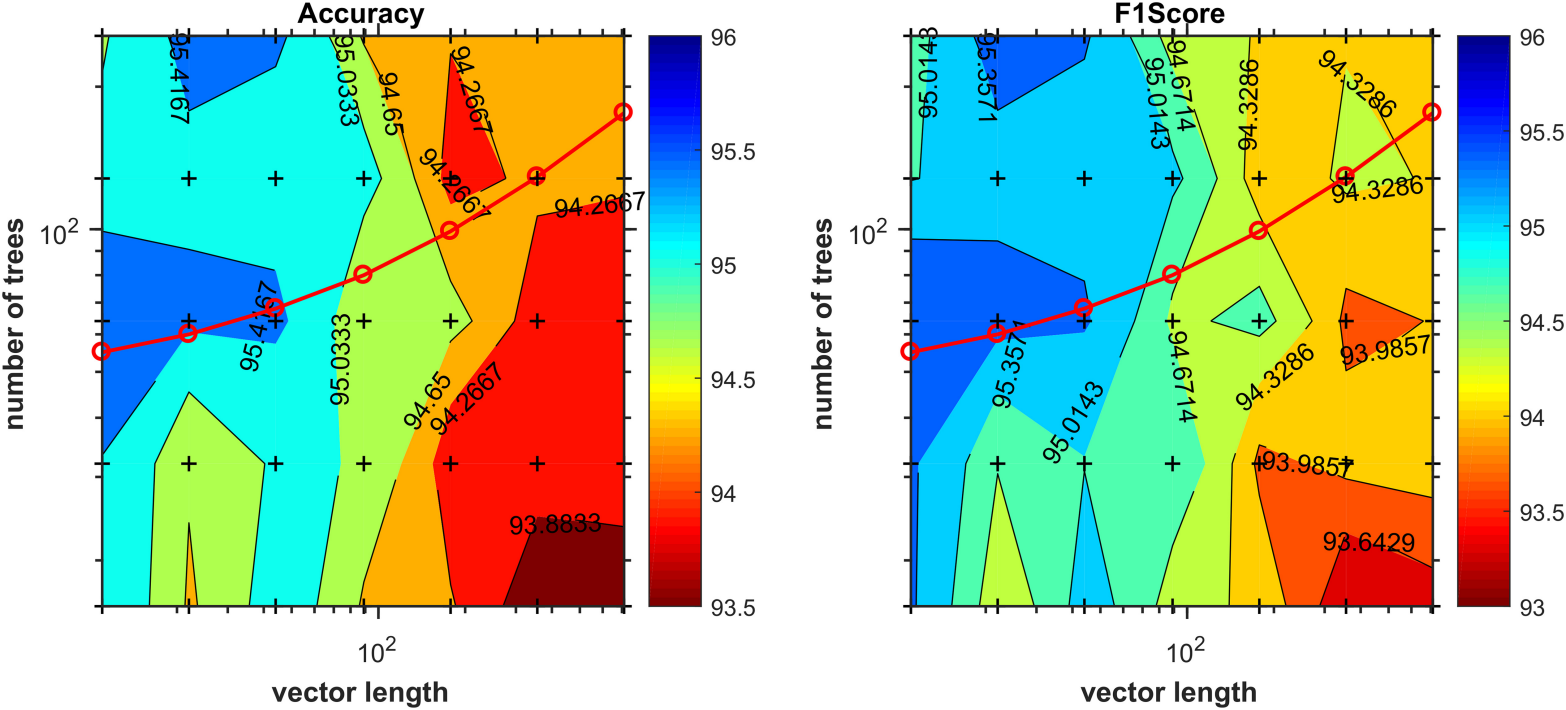
Number of trees given by the grid-search procedure as a function of vector length. Cross marks inside the chart denote values evaluated during the grid search. The red line corresponds to the function used later in the method to compute the number of trees during the training stage for the determination of most discriminant features.

**Figure 4:**
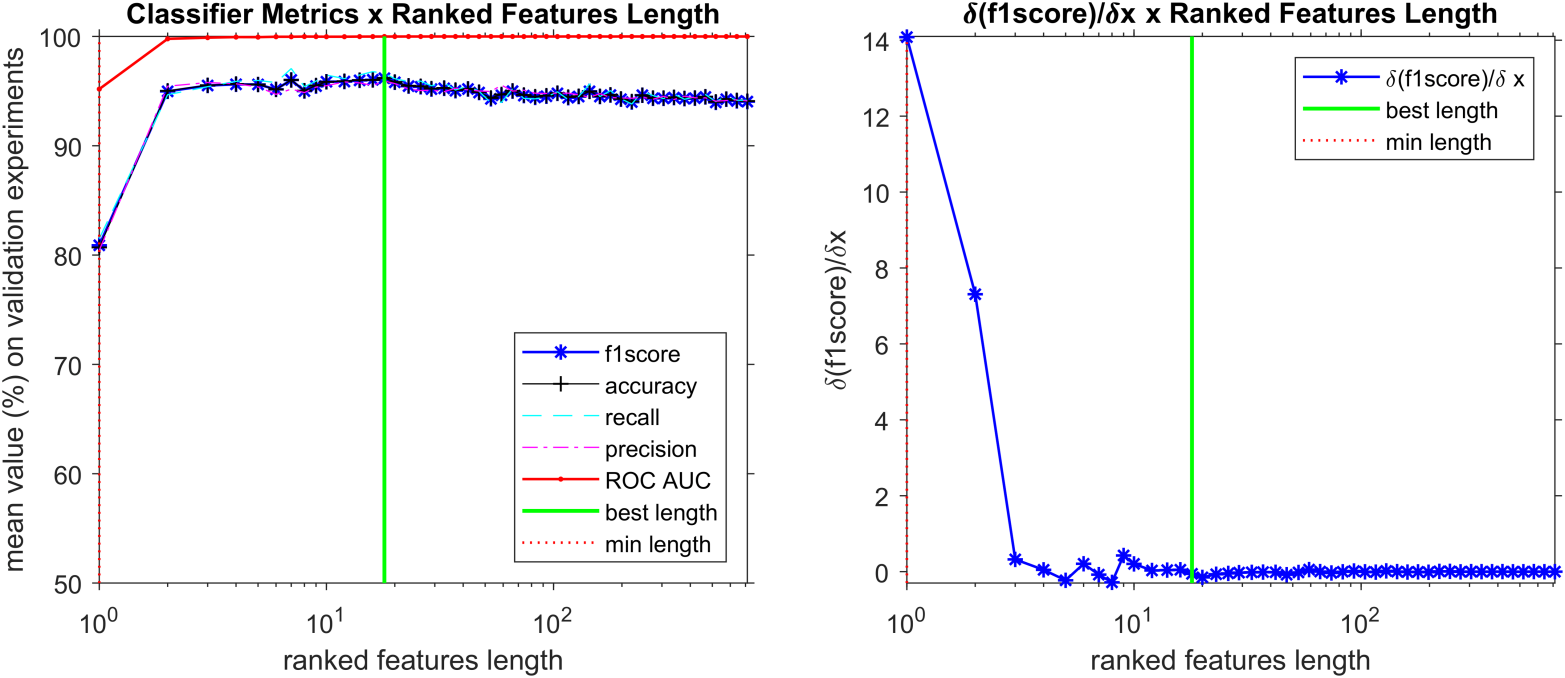
Search for most important discriminant features (ions), reducing the spectra data vector length while analyzing how f1-score is affected. The best classifier was found with 18 ranked features, including the 5 markers (discriminant features corresponding to ions that are more prevalent in positive samples) described in Table 4.

The appropriate hyperparameter *number of trees* in the Random Forest are shown in Figure 3, that depicts the plot of results of the grid search step, showing the curve (red line) that was selected to compute the number of trees in the further steps of the process. By looking at the chart on Figure 4, we found 18 features responsible for the maximum f1-score that represent good candidates for the metabolomics analysis.

Table 3 shows the ranked results for the 10 validation experiments using the optimal feature vectors and the Diagnosis Classifier final test results trained with ***P***_***fit***_ and tested with ***P***_***test***_ partitions respectively.

**Table 3:** Classification results of the validation tests and the final test of the Diagnosis Classifier using the 18 most discriminant features.

**Metric**	**Validation**		**Final test**
	**Mean**	**RSD**	
Vector length	18		18
Number of trees	58		58
Accuracy (%)	96.2	3.0	86.1
Sensitivity (%)	96.6	5.2	94.4
Specificity (%)	95.8	3.5	77.8
Precision (%)	95.9	3.3	81.0
F1-score (%)	96.2	3.2	87.2

**Figure 5:**
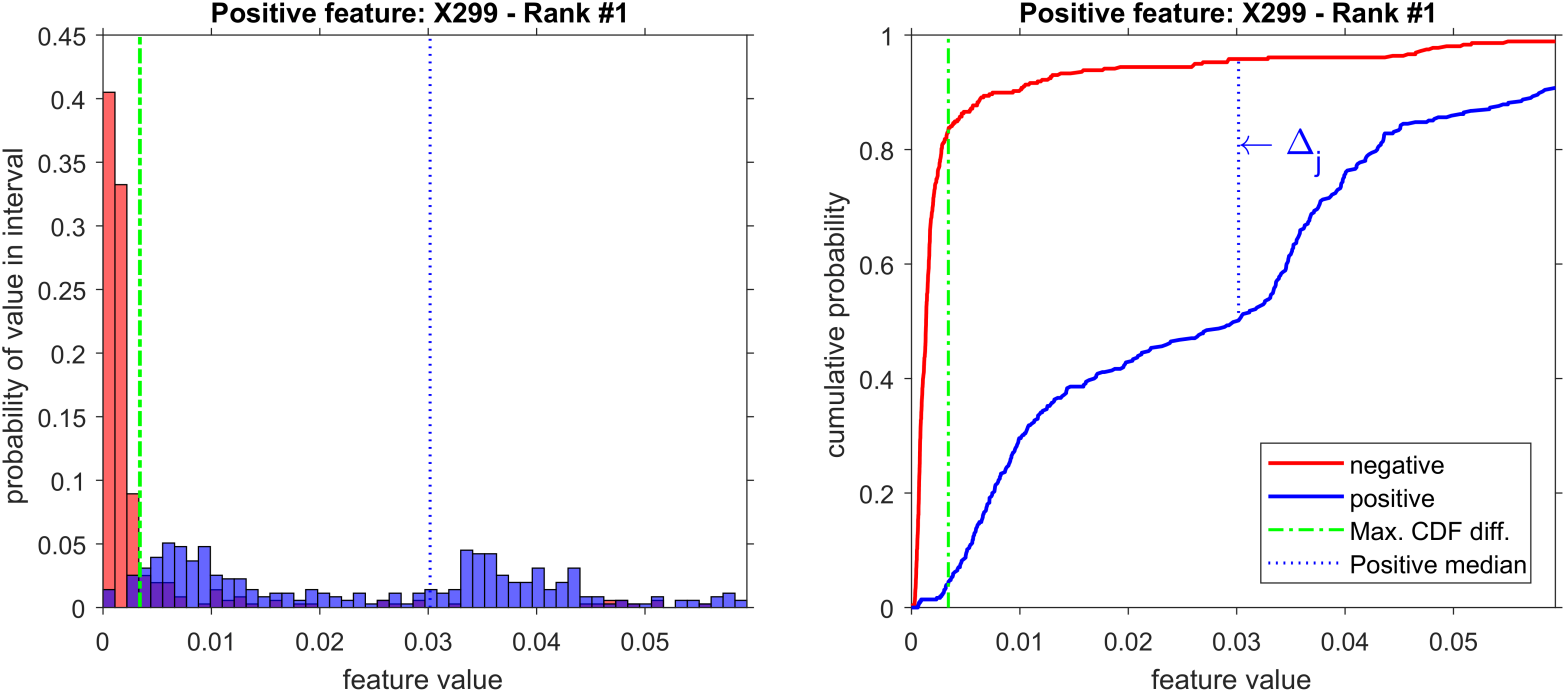
Distribution analysis of feature X299 (*m/z* = 299).

From the 18 most discriminant features, eight were identified as markers, as shown in Table 4. The first-ranked feature analysis is displayed on Figure 5. Distribution Analysis Chart for 18 Most Discriminant Features are displayed in Figure S1. Table 5 presents the ranked results when using only the markers features on the validation experiments, showing their capability to identify positive samples. Results of 18 most discriminant features are show in Table 6. All results are statistically comparable.

**Table 4:** The 18 most discriminant features found by the Random Forest analysis, highlighting the eight markers found herein.

**Rank**	**Marker**	***m/z***	**Rank**	**Marker**	***m/z***
1	Yes	299	10	Yes	283
2	No	673	11	Yes	389
3	Yes	278	12	No	252
4	No	672	13	No	656
5	Yes	263	14	No	395
6	No	379	15	Yes	270
7	Yes	304	16	No	315
8	No	657	17	No	462
9	No	250	18	Yes	308

**Table 5:** Results of the 10 experiments validation with Random Forest classifiers trained with the eight markers.

**Metric**	**Mean**	**RSD**
Vector length	8	
Number of trees	58	
Accuracy (%)	90.9	4.0
Sensitivity (%)	93.5	3.9
Specificity (%)	88.4	7.3
Precision (%)	89.5	6.2
F1-score (%)	91.3	3.6

**Table 6:** Comparison of validation results of 18 most discriminant features using different classifiers.

**Metric**	**SVM (SMO)**		**SVM (ISDA)**		**Random Forest (GDI)**		**Tree (GDI)**	
	**Mean**	**RSD**	**Mean**	**RSD**	**Mean**	**RSD**	**Mean**	**RSD**
Accuracy (%)	96.6	2.3	97.2	2.3	96.5	3.3	96.2	3.5
Sensitivity (%)	97.4	4.5	98.6	3.0	97.1	5.0	95.9	5.7
Specificity (%)	95.8	5.0	95.8	5.0	95.8	4.8	96.5	3.7
Precision (%)	96.2	4.3	96.2	4.3	96.0	4.7	96.6	3.7
F1-score (%)	96.6	2.2	97.3	2.1	96.5	3.5	96.1	3.7

*SVM with two different optimization algorithms: SMO, Sequential Minimal Optimization (Fan et al., 2005); ISDA, Iterative Single Data Algorithm (Kecman et al., 2005), and single decision tree classifier using (GDI) Gini Diversity Index (Breiman et al., 1984) as the split criterion. Green font means best classifier and red font worst classifier for each metric (table line)*.

### Biomarkers Characterization

Thereafter, the eight markers identified underwent metabolomics techniques to identify which ones are weight gain biomarkers, as described next herein. After checking the exact masses in the metabolomics databases and literature, five biomarkers were characterized: Dihydrobiopterin, Argininosuccinate, 3-carboxy-4-methyl-5-propyl-2-furanpropanoic acid (CMPF), a prostaglandin (PGB2) and a metabolite of leukotriene (Carboxy-LTB4). The description of identified markers is listed in Table 7. The Heatmap analysis distribution of 5 biomarkers identified is displayed in Figure S2.

**Table 7:** Markers elected by Random Forest from plasma analysis of case group.

**Nominal mass**	**Exact mass**	**Mass fragments**	**Adduct**	**Theoretical mass**	**Error (ppm)**	**Metlin ID**	**Molecule**
278	278.0655	232–260–236–246	[M+K]^+^	278.0650	1.79	65,872	Dihydrobiopterin
299	299.2022	263–271–213–281	[M+H-2H_2_O]^+^	299.2017	1.67	3,466	PGB2
308	308.1571	290–248–209	[M+NH_4_]^+^	308.1565	1.94	389	Argininosuccinate
389	389.1942	353–319–371–285	[M+Na]^+^	389.1935	1.79	36,247	Carboxy-LTB4
263	263.0885	245 [227^*^]−165	[M+Na]^+^	263.0890	−1.90	45,041	CMPF

* *MS3*.

With the support of previous studies, it was possible to demonstrate a link between weight gain and systemic inflammation, and describe the roles of biomarkers in the pathways associated to an imbalance in the Nitric Oxide (NO) cycle and the overweight status-related metabolic stress.

## Discussion

Upon elucidation of weight gain-associated biomarkers, it was possible to demonstrate the presence of Argininosuccinate [*m/z* 308.1565], a basic amino acid and immediate precursor of arginine in the urea cycle. Numerous studies have shown a relationship between arginine, obesity and other metabolic disturbance (Pallares-Méndez et al., 2016; Zhang et al., 2017), but its exact role and related metabolic pathways still require a more robust amount of studies (Lent-Schochet et al., 2019). Argininosuccinate is synthesized from the condensation of citrulline and aspartate by the enzyme argininosuccinate synthase; then, it is cleaved into fumarate and arginine by the enzyme argininosuccinate lyase (Haines et al., 2011). Interestingly, according to studies carried out with rodents, argininosuccinate may actively participate in the shift of M2 macrophages to M1 in adipose tissue, indicating that this molecule is a potential marker of activation of proinflammatory macrophages in human obesity as well (Fraternale et al., 2015; Jha et al., 2015; Kuda et al., 2016).

Dihydrobiopterin (BH_2_) [*m/z* 278.0650] is a product of the oxidation of Tetrahydrobiopterin (BH_4_), an important cofactor for NO synthase (NOS). Whenever there is a balance between cofactors and substrates in relation to Nicotinamide adenine dinucleotide phosphate (NADPH), NOS catalyzes the reduction of O_2_ and incorporates it into the guanidine group of L-arginine, generating NO. Nonetheless, in specific situations, such as the oxidation of BH_4_ into BH_2_, a molecule that cannot work as a cofactor for NOS, a phenomenon called “uncoupling” occurs. Uncoupled NOS generates superoxide instead of nitric oxide, leading to an oxidative stress state. Further, produced reaction oxygen species (ROS) may oxidize BH_4_ into BH_2_, exacerbating this state (Incalza et al., 2018). It is known that reactive oxygen species may induce and increase the inflammatory process by upregulating different genes involved in the inflammatory response, such as those that induce the production of cytokines and adhesion molecules (Galvão et al., 2018). One of the effects of ROS on cells is the modification of fatty acids from the phospholipid membrane, a process that alters membrane fluidity and cell signaling. In oxidative stress, the enzyme phospholipase A presents increased activity, producing active mediators such as arachidonic acid (AA) metabolites (Balboa and Balsinde, 2006).

AA metabolites, namely Eicosanoids, are a family of lipid mediators generated from phospholipid precursors, involved in distinct cellular processes. In an inflammatory environment, the enzyme Phospholipase A2 catalyzes the esterified AA present in the phospholipid membrane into free AA, which may be then oxidized in four different pathways: lipoxygenase (LOX), cyclooxygenase (COX), epoxygenase (CYTP450) and isoprostane. Prostaglandins are produced through the COX pathway, and leukotrienes through the LOX pathway. Two markers found in the plasma of the case group are AA metabolites: Prostaglandin B2 [*m/z* 299.2017] and Carboxy-Leukotriene B4 [*m/z* 389.1935], indicating that at least two AA oxidation pathways are putatively more active in the case group.

The most studied Prostaglandin, with the highest pro-inflammatory effects described in prior art, is PGE2. The biomarker found herein is obtained from the dehydration of PGE2 and has a number of studies describing its effects. In a study with cell cultures, Cattan et al. evaluated the regulation of different prostaglandins in an inflammation site. The main finding was that PGB2 induced Interleukin 2 (IL-2), activation of tyrosine kinase activities, and Nuclear factor kappa B (NF-κB). According to the authors, PGB2 behaves as a potent lipid mediator and mimics almost all effects of PGE2 (Cattan et al., 2000).

LTB4, the precursor of the other biomarker involved with AA pathways in this study, is a potent lipid mediator capable of promoting chemotaxis, neutrophil degranulation, and release of enzymes and ROS. LTB4 has a receptor with high specificity, B leukotriene receptor 1 (BLT1), expressed exclusively in inflammatory cells such as neutrophils, macrophages and eosinophils. *In vivo*, LTB4 is rapidly metabolized by different pathways in an attempt to decrease the inflammatory effects caused by this mediator. From neutrophil action, and with the participation of ω-hydroxylase, LTB4 is oxidized to 20-carboxy LTB4. The oxidized products of LTB4 were believed to be inactive species, but binding studies demonstrated that 20-carboxy LTB4 has the ability to bind to LTB1 and had the same chemotactic effects, suggesting a similar action of this molecule on inflammation (Wang et al., 2000; Toda et al., 2002).

Given the fact that these AA-derived metabolites are closely associated with inflammation, and since this study was not tissue-specific, their presence reinforce the concept that the inflammatory activity associated with obesity occurs systemically. This result agrees with several previous studies that have demonstrated the relation of obesity to other complications and diseases in several organs, such as insulin resistance, type 2 diabetes, dyslipidemia, and disorders in immune function (Doupa et al., 2014; Finn, 2015; Kohlgruber and Lynch, 2015; Velloso et al., 2015).

A number of studies published previously have shown alterations in mitochondrial activity, NO cycle, and increase of reactive oxygen species at the onset of obesity, including childhood and juvenile obesity (Williams et al., 2002; Gruber et al., 2008). According to Muñoz and Costa, the increase in glucose and free fatty acids from energy imbalance causes an increase in the production of Acetyl-Coenzyme A (Acetyl-CoA) in the Tricarboxylic Acid (TCA) cycle, which stimulates the excessive production of superoxide and, consequently, the activation of inflammatory stimuli (Muñoz and Costa, 2013). In a study that evaluated gene expression and cytokine dosage in plasma and tissue before and after induction of obesity in animals fed a high-fat diet, Matsuzawa-Nagata et al. demonstrated that up-regulation of ROS gene expression preceded the onset of obesity, and also the production of Tumor Necrosis Factor alpha (TNF-α), suggesting that the onset of obesity is related to an imbalance in ROS production (Matsuzawa-Nagata et al., 2008).

**Figure 6:**
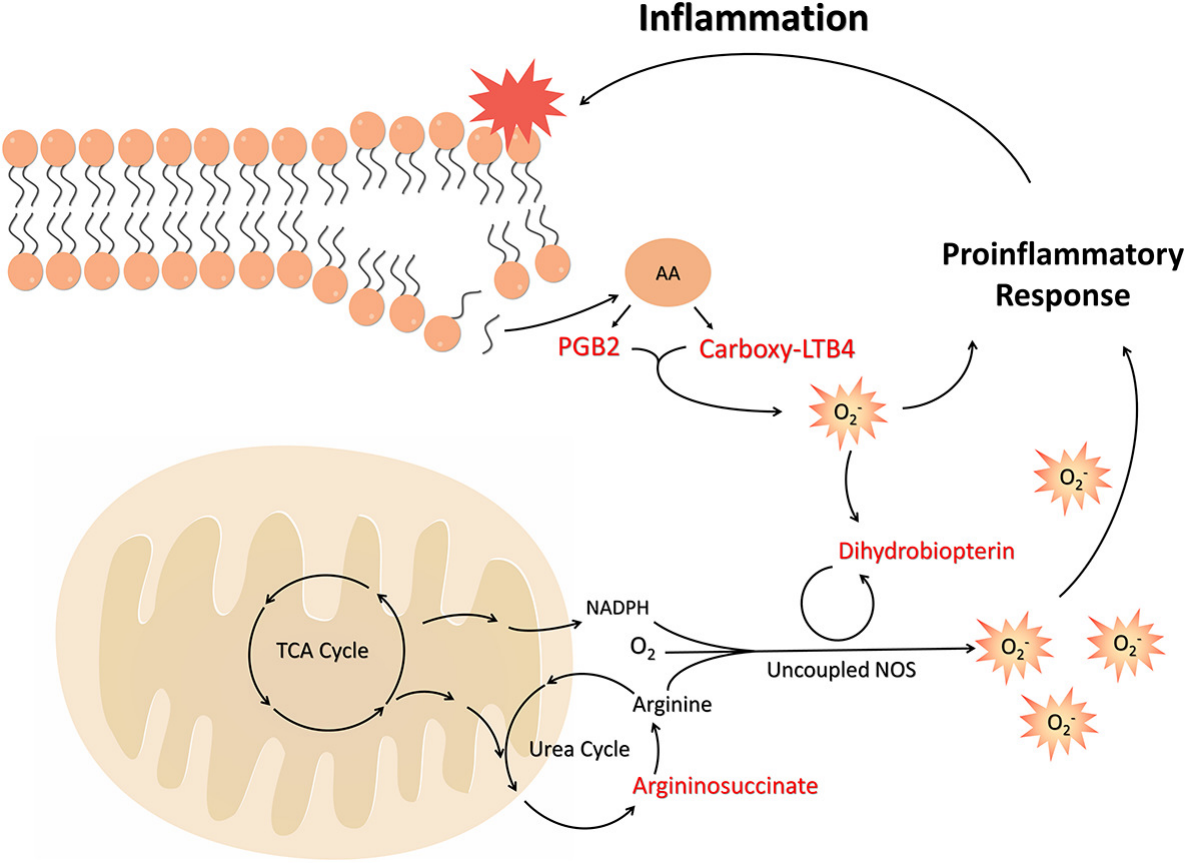
The unbalance of cofactors and substrates in the NO cycle, along with the oxidation of tetrahydropterin to dihydrobiopterin, leads to the uncoupled effect and increased superoxide production. This state of oxidative stress leads to the induction and increase of inflammatory mediators, such as arachidonic acid metabolites. These, in turn, are able to induce ROS and oxidation of TH4, exacerbating the inflammatory state and generating its chronicity in obesity. FFA, Free Fat Acids; TCA, Tricarboxylic Acid; NADPH, Nicotinamide adenine dinucleotide phosphate; NOS, Nitric Oxide Synthase; AA, Arachidonic acid; PGB2, Prostaglandin B2; Carboxy-LTB4, Carboxy-Leukotriene B4.

Considering that obesity is related to the increase of Acetyl-CoA combined with disturbances in the NO cycle and consequent activation of inflammatory mediators from the release of ROS, the biomarkers found are actively involved in the process of onset of obesity and development of chronicity of low-grade inflammation in a pathway described in Figure 6.

Finally, another marker found was 3-carboxy-4-methyl-5-propyl-2-furanpropanoic acid (CMPF) [*m/z* 263.0890], a dibasic urofuran acid catabolized from furan fatty acids incorporated into phospholipids and cholesterol esters, already described in the literature as related to uremia. Recent studies have found a relationship of this metabolite with a beta cell dysfunction and the development of diabetes (Prentice et al., 2014; Liu et al., 2016). Considering that there were diabetic patients within our case group, further studies are needed to attest whether this molecule may also be related to obesity.

Several studies have sought to elucidate biomarkers through metabolomic techniques in the contexts of obesity, diabetes, cardiovascular diseases, and MetS. The main purposes supporting these contributions are to broaden the understanding on the metabolic environment of individuals in these different conditions, as well as potential molecular targets that link them all (Newgard, 2017). Studies have already shown the relationship between cardiometabolic diseases, obesity and diabetes with disturbances in the amino acid pathways such as aromatics and branched-chain amino acids (BCAA) (Newgard, 2017; Rauschert et al., 2017; Zhong et al., 2017; Libert et al., 2018; Del Coco et al., 2019).

In an extensive review of the literature, Zhang et al. have collected several studies that associate obesity with markers present in different metabolic pathways, such as lipids, lysophosphatidylcholines, monosaccharides, acylcarnitines, and metabolites related with TCA cycle, tryptophan, phenylalanine, and tyrosine pathways (Zhang et al., 2017). In a study conducted in Australia, Huynh et al. used robust lipidomics techniques to determine markers related to cardiometabolic risk factors and anthropometric measures in human plasma. According to the study, there are 338 plasma lipid species related with BMI, being positively associated species such as sphingomyelin, phosphatidylcholine, ceramides, and phosphatidylethanolamine (Huynh et al., 2019).

Our results also demonstrate that the field of metabolomics can contribute comprehensively to the study of the onset of obesity and its development. From the plasma of case patients, with the metabolomics strategies combined with advances in data processing using machine learning, it was viable to elect biomarkers of obesity and describe a possible pathway of inflammation in this and other associated comorbidities. This study contributes with knowledge on aspects of the relationship between obesity, systemic inflammation and chronicity. Our approach—contribute for elucidation of potential therapeutic targets in obesity and other disorders and pathologies. Despite that metabolomics is a sophisticated method, that requires expensive equipment and trained personnel, the simplicity in sample preparation, short time to analyze metabolites, and the use of modern data processing techniques for the determination of obesity biomarkers are the new insights proposed in this study. Finally, it also collaborates with upcoming studies in the fields of prevention and diagnosis of obesity.

## Data Availability Statement

All data will be available in MetaboLights accession MTBLS1400 as soon as the curation process at Metabolights is finished. Thus, we cannot provide anything immediately, as at this stage our actions are all complete and we depend on the system workflow; we expect, however, that the process will be over within the next couple of weeks.

## Ethics Statement

The studies involving human participants were reviewed and approved by Human Ethics Committee of the State University of Londrina, Paraná, Brazil. The patients/participants provided their written informed consent to participate in this study.

## Author Contributions

FD-A and DO performed mass spectrometry experiments. LN and AR conceived and executed the ML method. FD-A, DO, and LN wrote the manuscript. FD-A, DO, TG, CM, and JD performed data analysis. FD-A, DP, FR, and MW performed patient recruitment, biofluids management, and clinical support. FD-A and JD processed serum samples. FD-A, LN, DO, LV, AR, and RC performed manuscript proofreading and prepared tables and figures. RC idealized all experiments and managed the research group.

### Conflict of Interest

The authors declare that the research was conducted in the absence of any commercial or financial relationships that could be construed as a potential conflict of interest.
