# Supplementary material for: Combining Machine Learning and Metabolomics to Identify Weight Gain Biomarkers
Source: Front Bioeng Biotechnol. 2020 Jan 24;8:6. doi: 10.3389/fbioe.2020.00006 (PMC6993102; doi:10.3389/fbioe.2020.00006)
Supplement: Supplementary file 1 [file Table_1.docx]

Combining machine learning and metabolomics to identify weight gain biomarker

**Flávia Luísa Dias-Audibert¹†, Luiz Claudio Navarro²** **†, Diogo Noin de Oliveira¹, Jeany Delafiori¹, Carlos Fernando Odir Rodrigues Melo¹, Tatiane Melina Guerreiro¹, Mohamed Ziad Dabaja¹, Flávia Troncon Rosa³, Diego Lima Petenuci^4^, Maria Angelica Ehara Watanabe^4^, Licio Augusto Velloso^5^, Anderson Rezende Rocha²*, Rodrigo Ramos Catharino¹***

1 Innovare Biomarkers Laboratory, School of Pharmaceutical Sciences, University of Campinas, Campinas, Brazil

2 RECOD Laboratory, Institute of Computing (IC), University of Campinas, Campinas, Brazil

3 Centro Universitário Filadélfia, Londrina, Brazil

4 Laboratory of Studies and Applications of DNA Polymorphisms, Biological Sciences Center, Londrina State University, Londrina, Brazil

5 Department of Internal Medicine, School of Medical Sciences, University of Campinas, Campinas, Brazil.

Supplemental Material

**(a)**


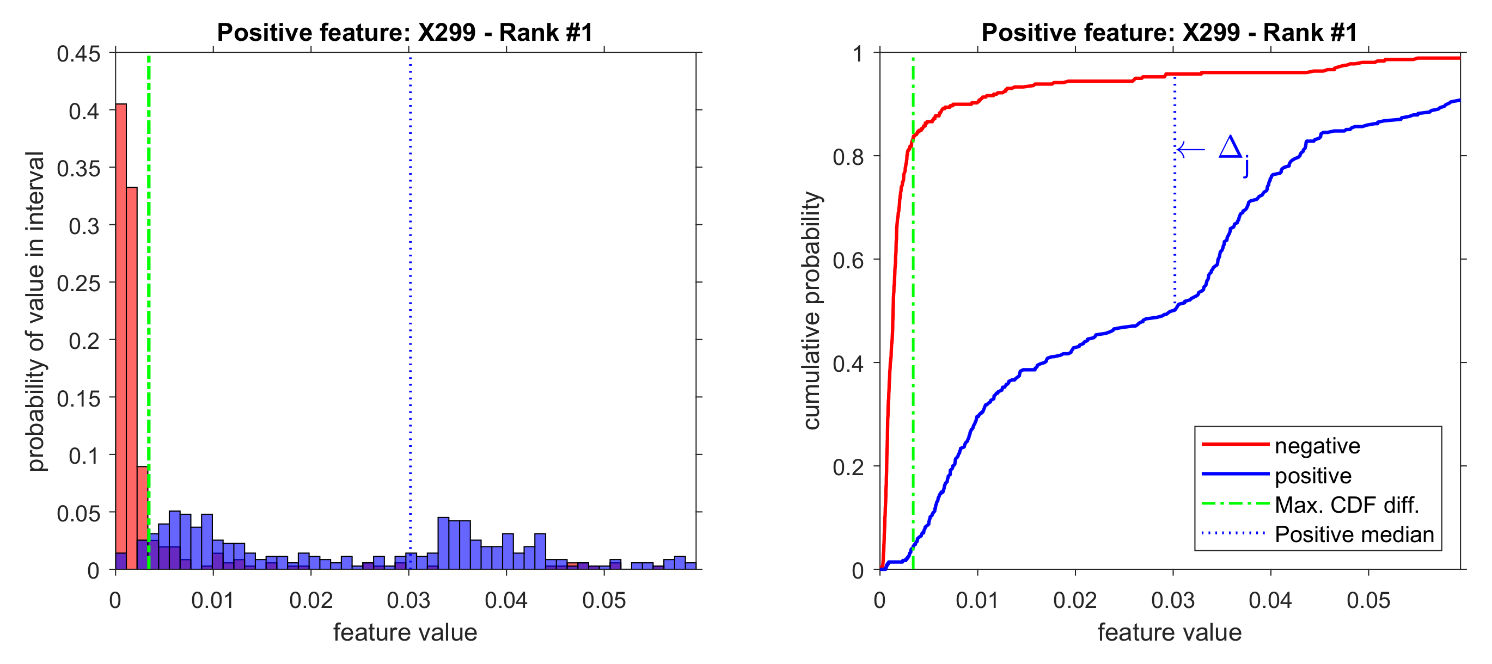

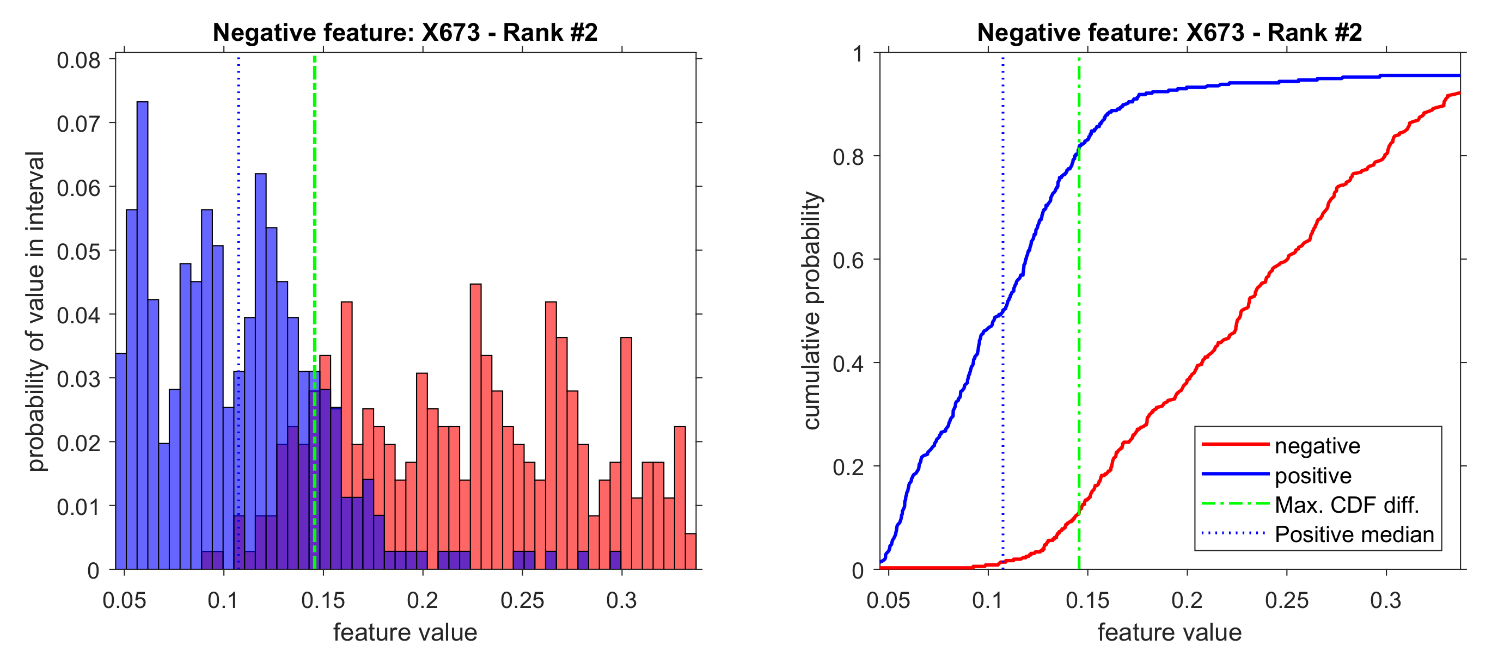

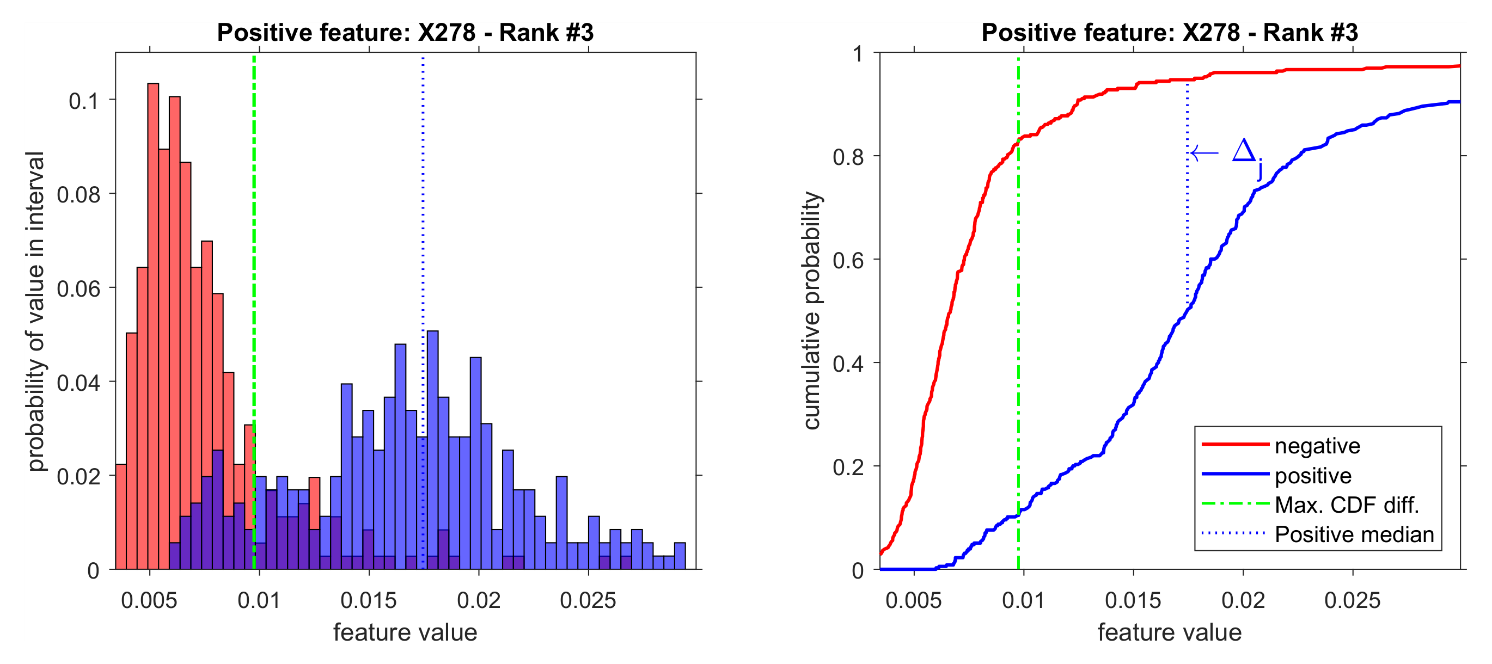

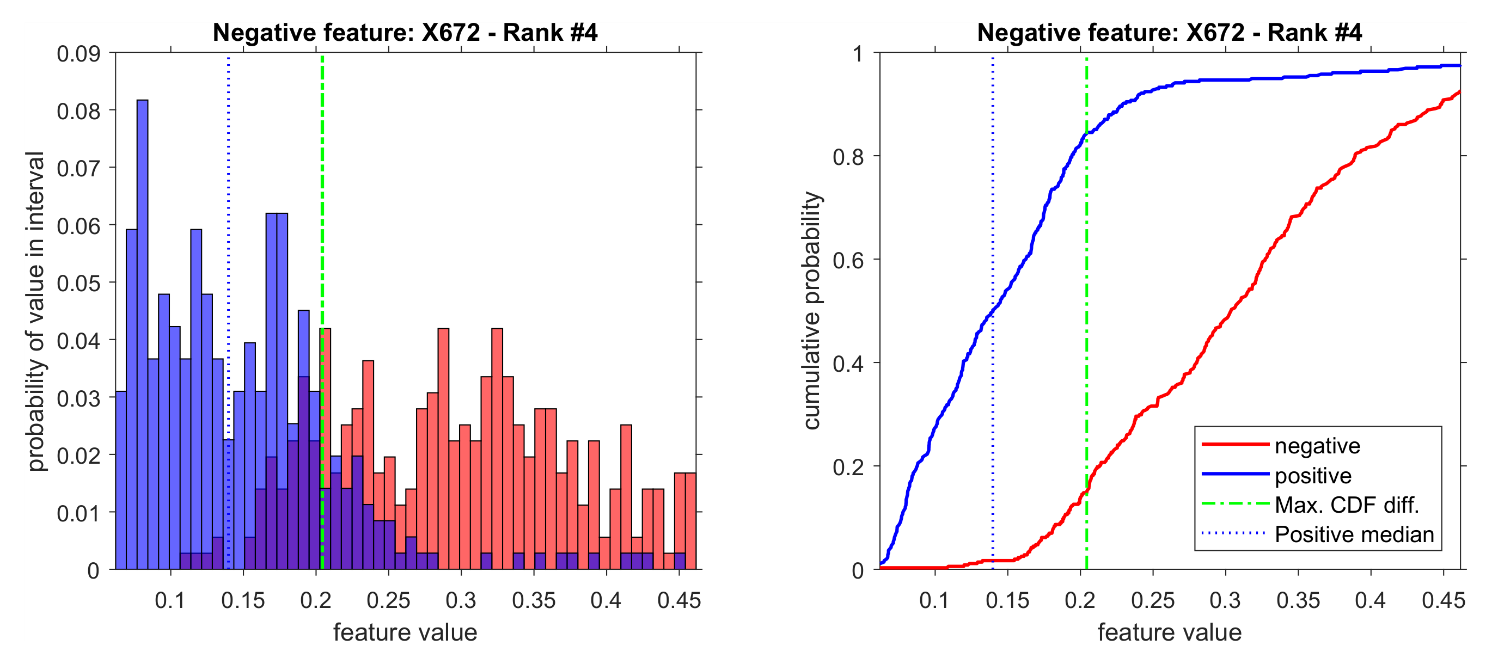

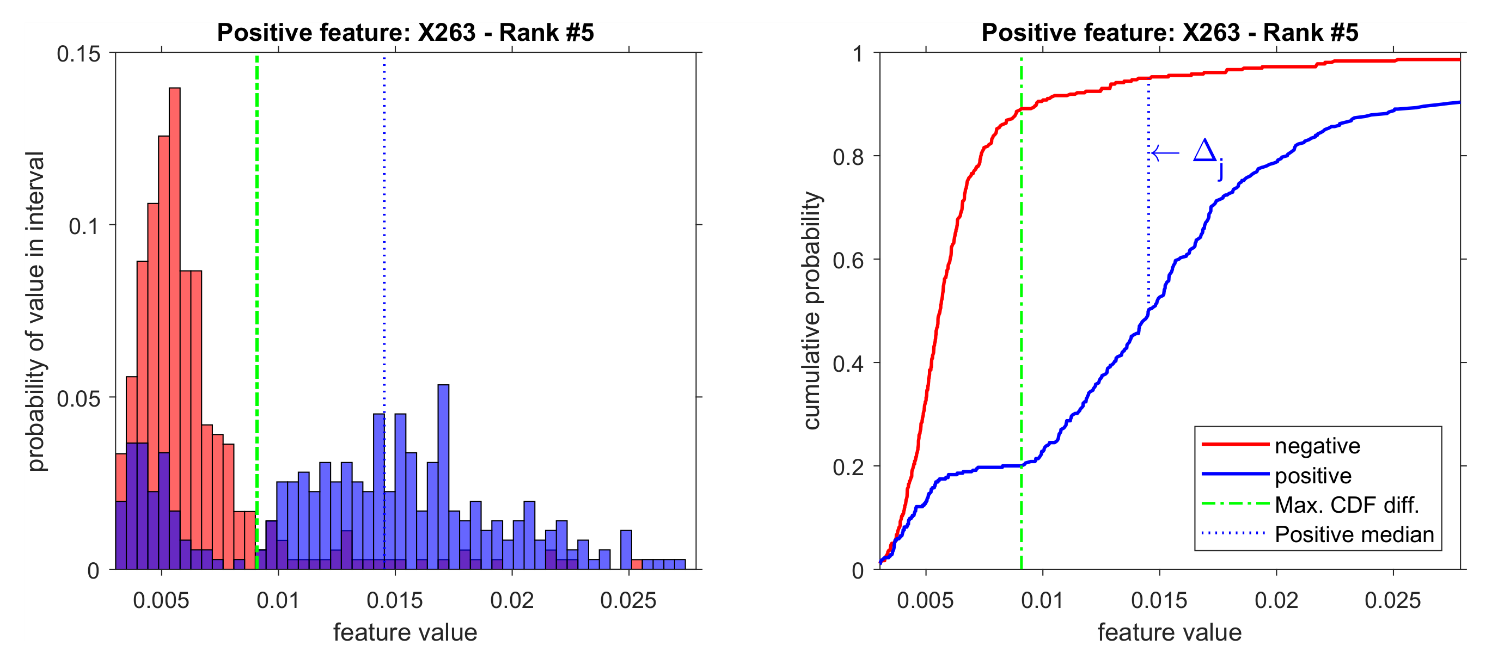

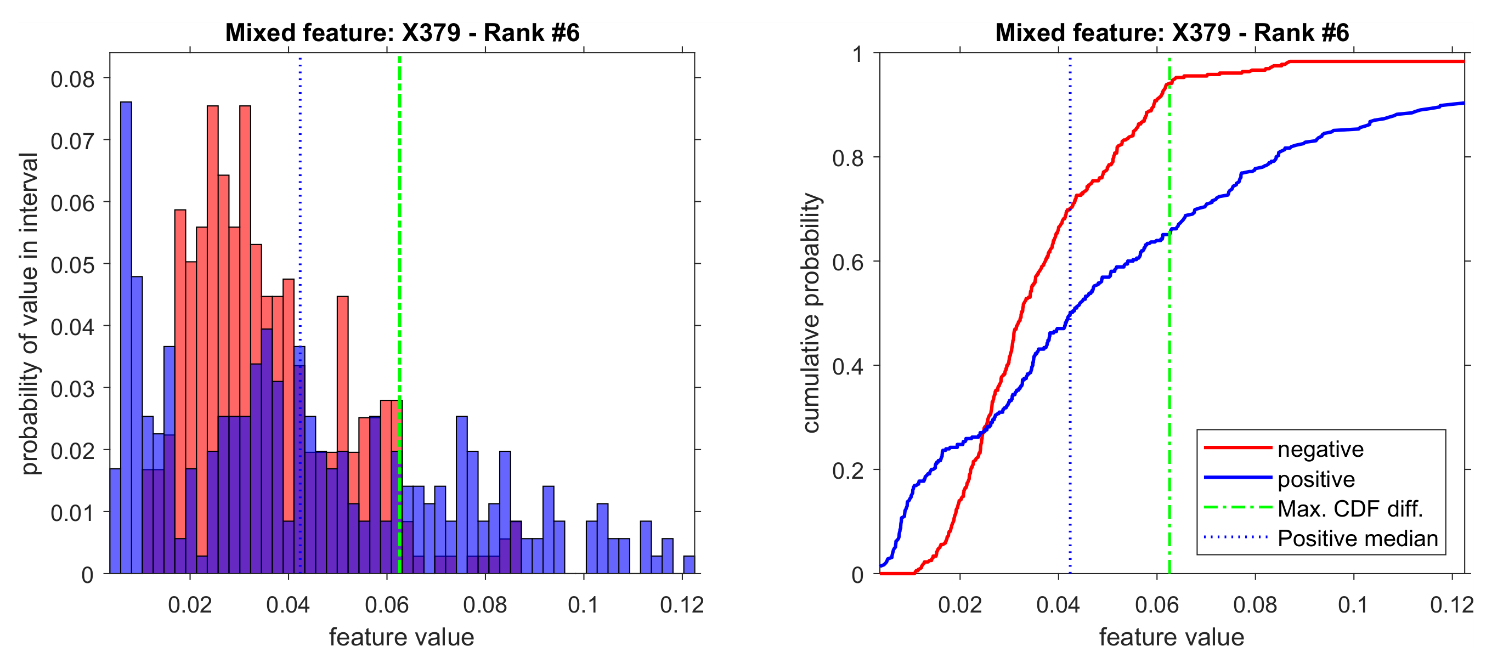

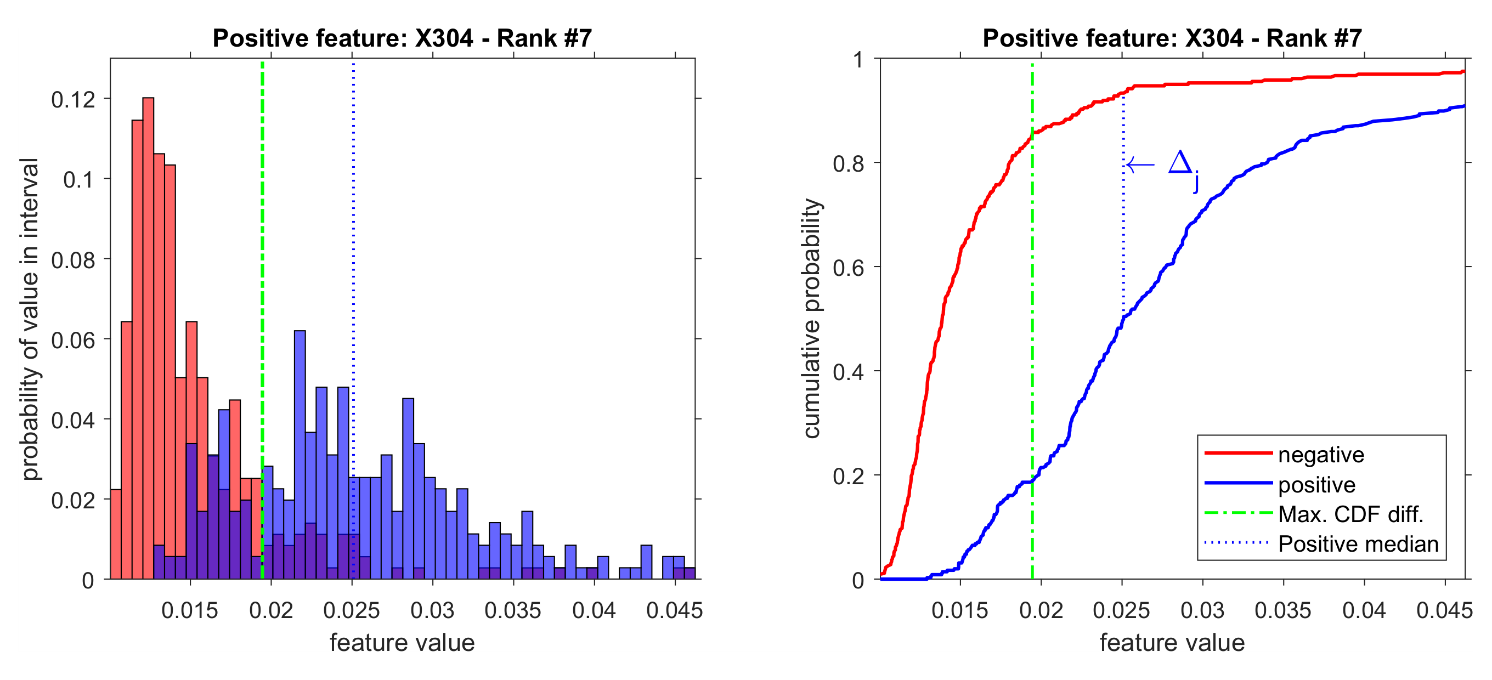

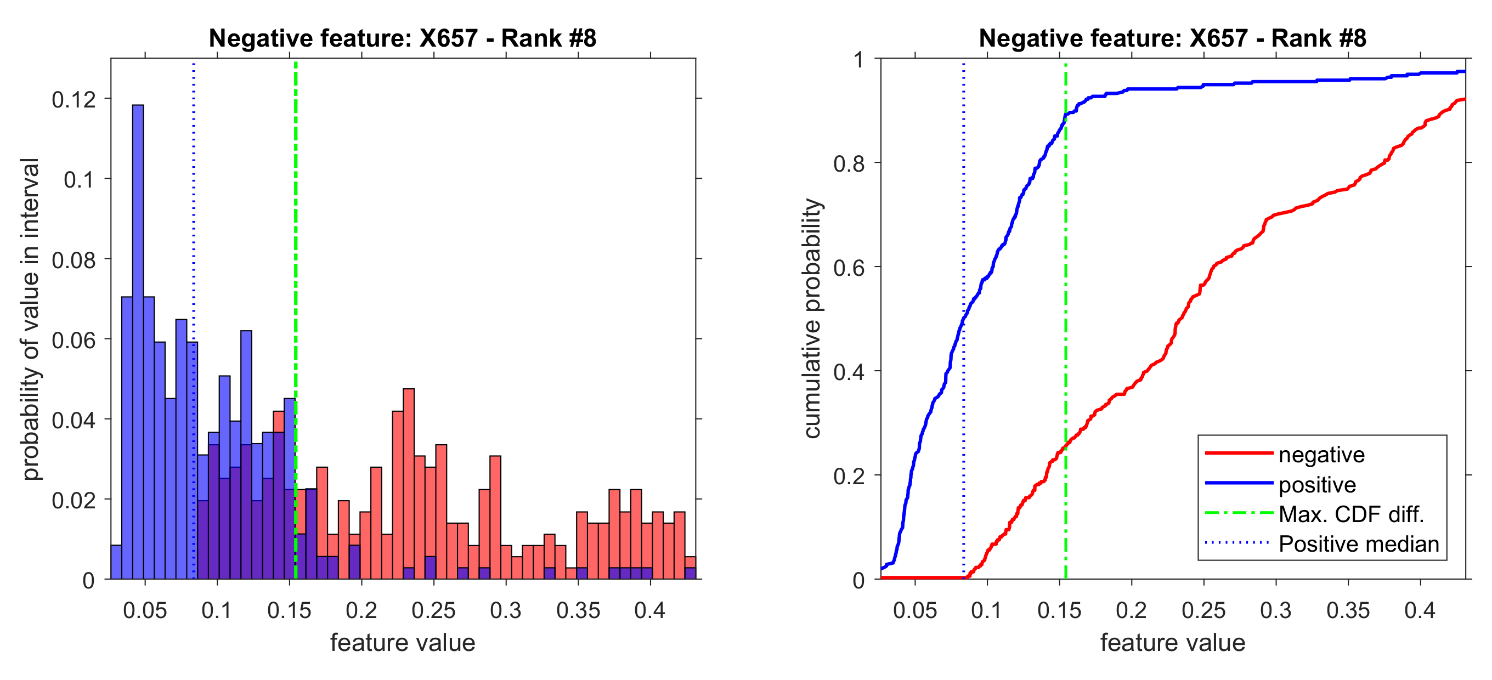

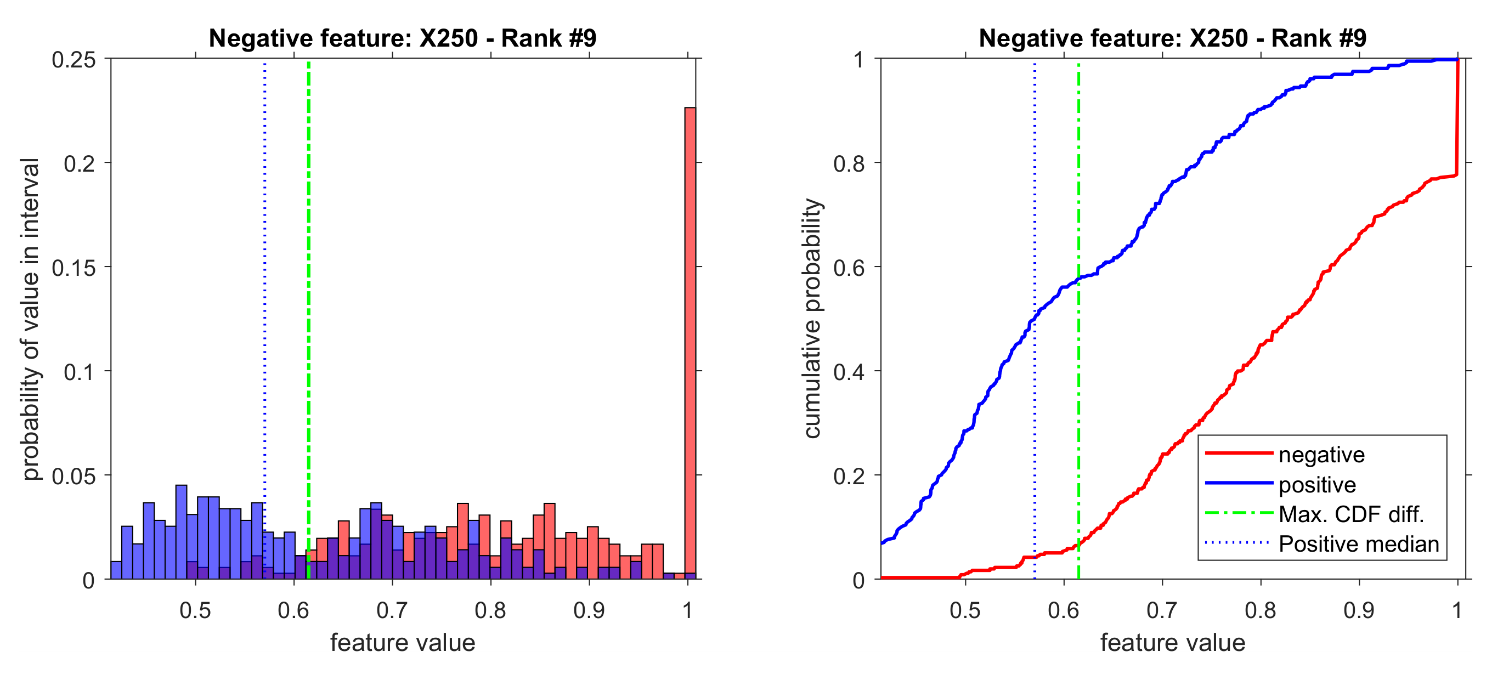

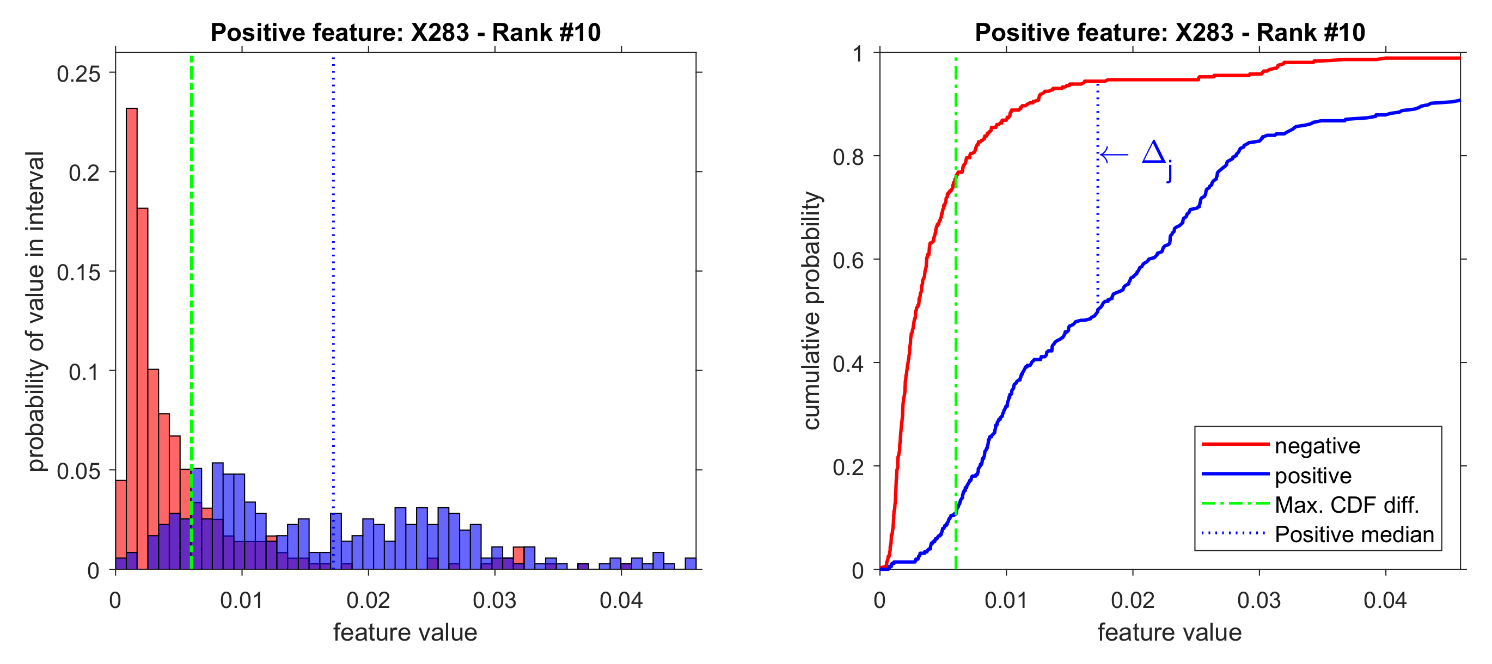

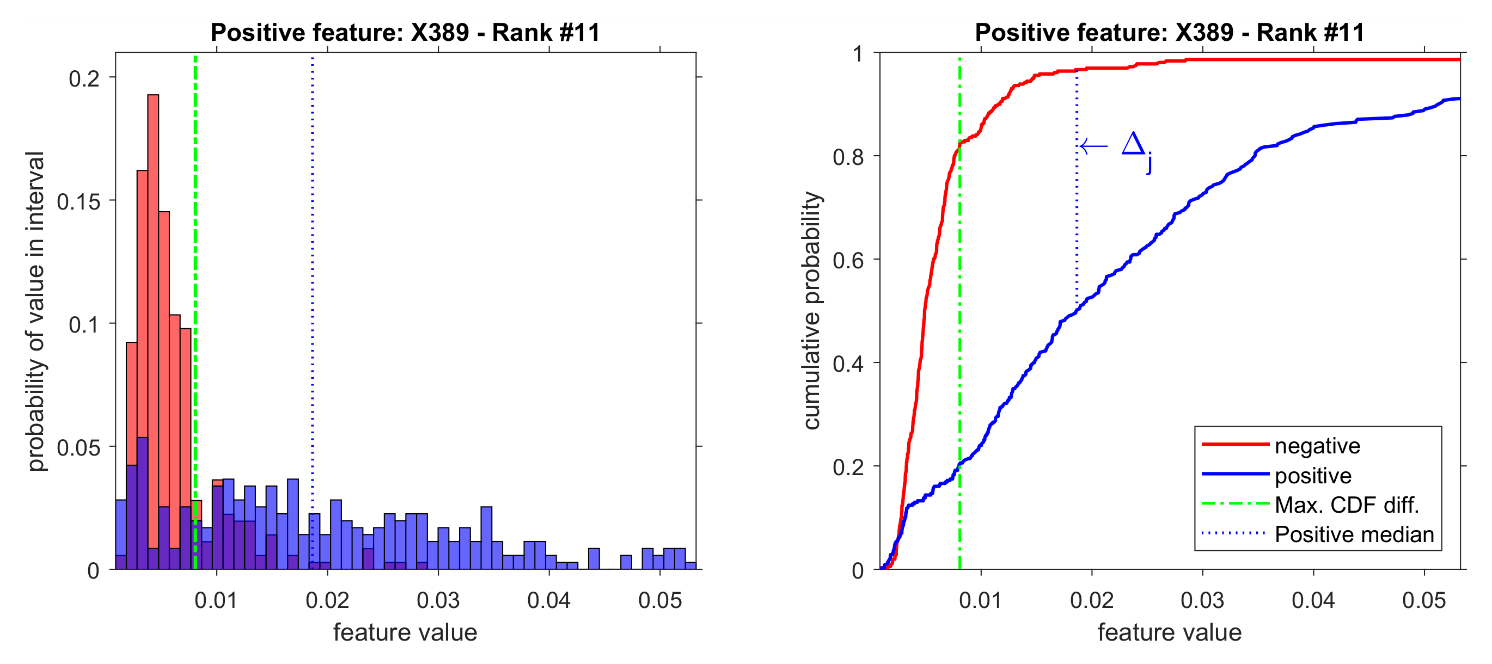

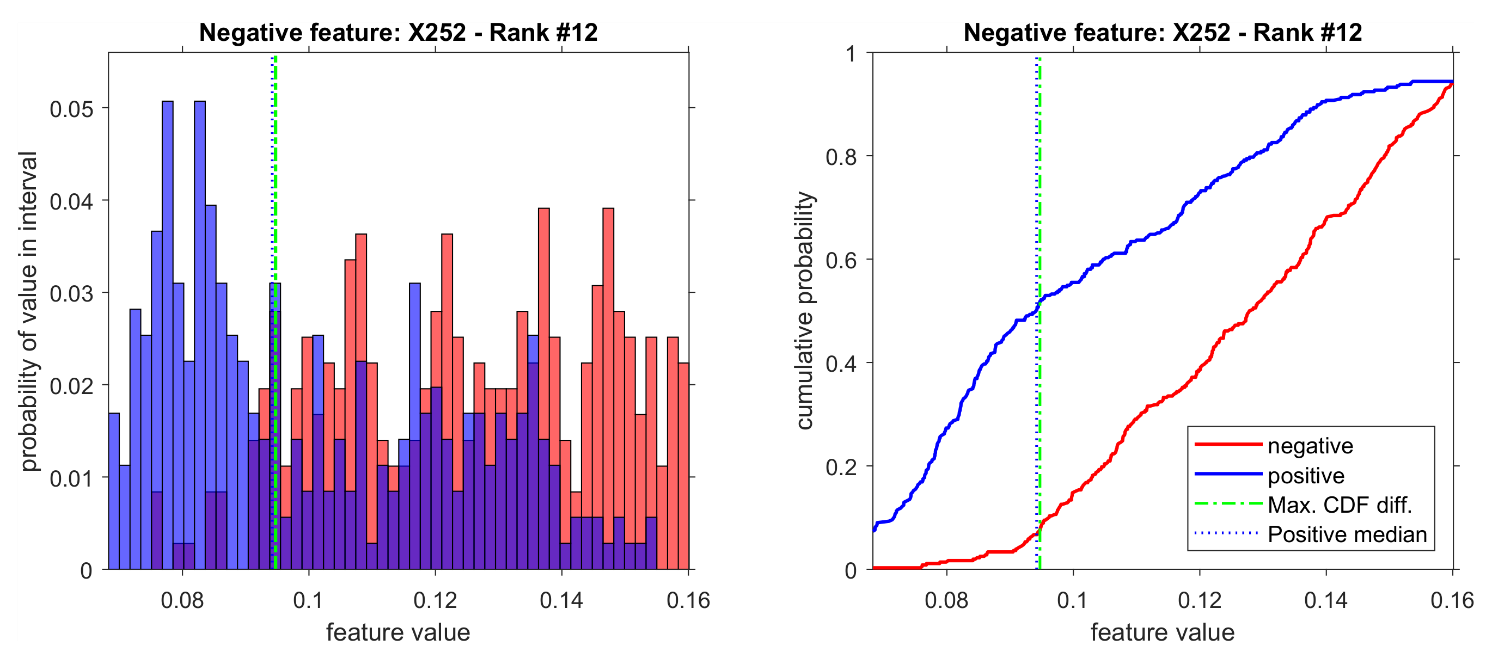

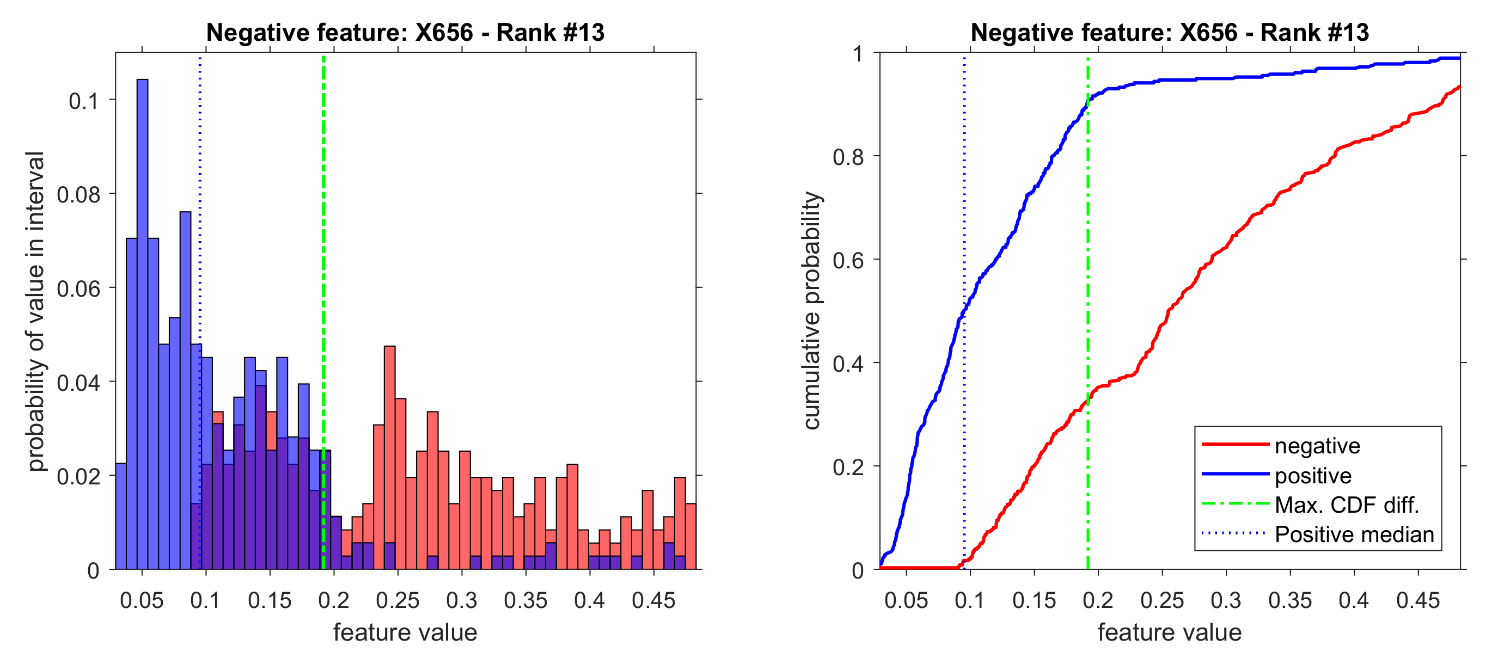

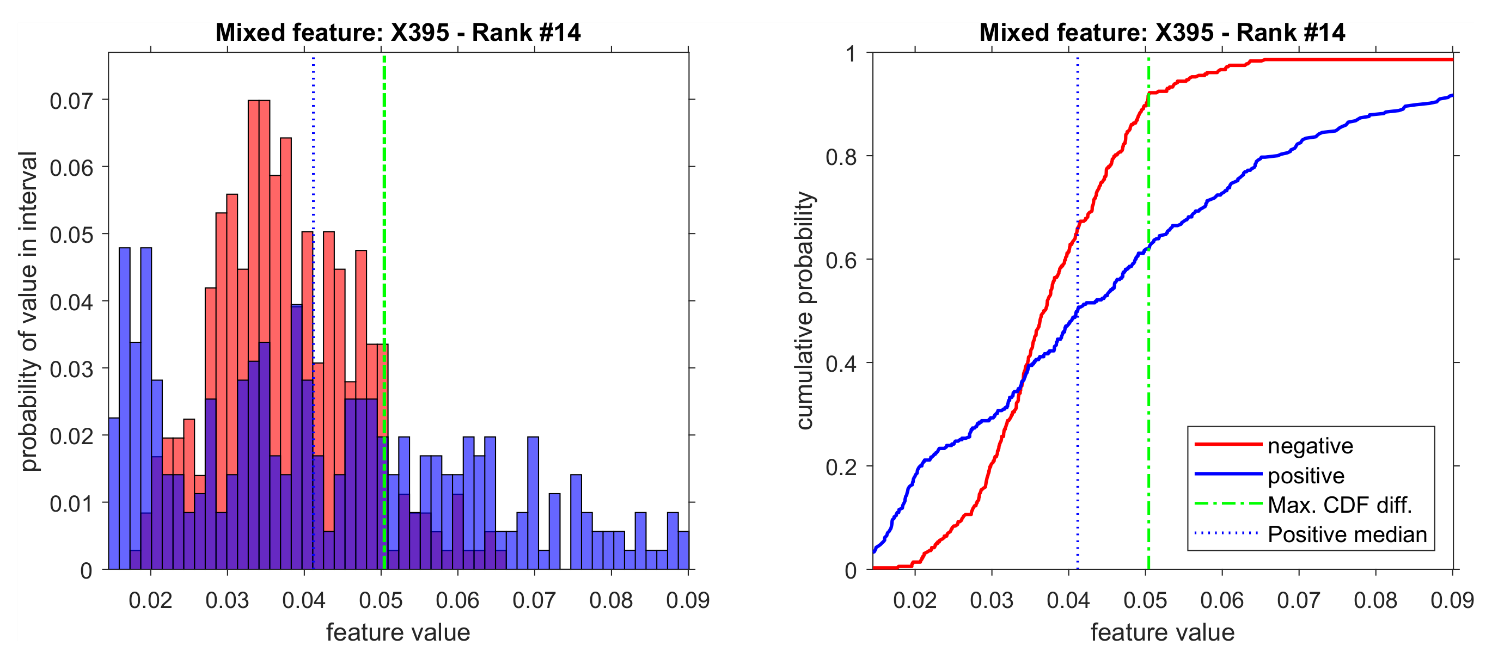

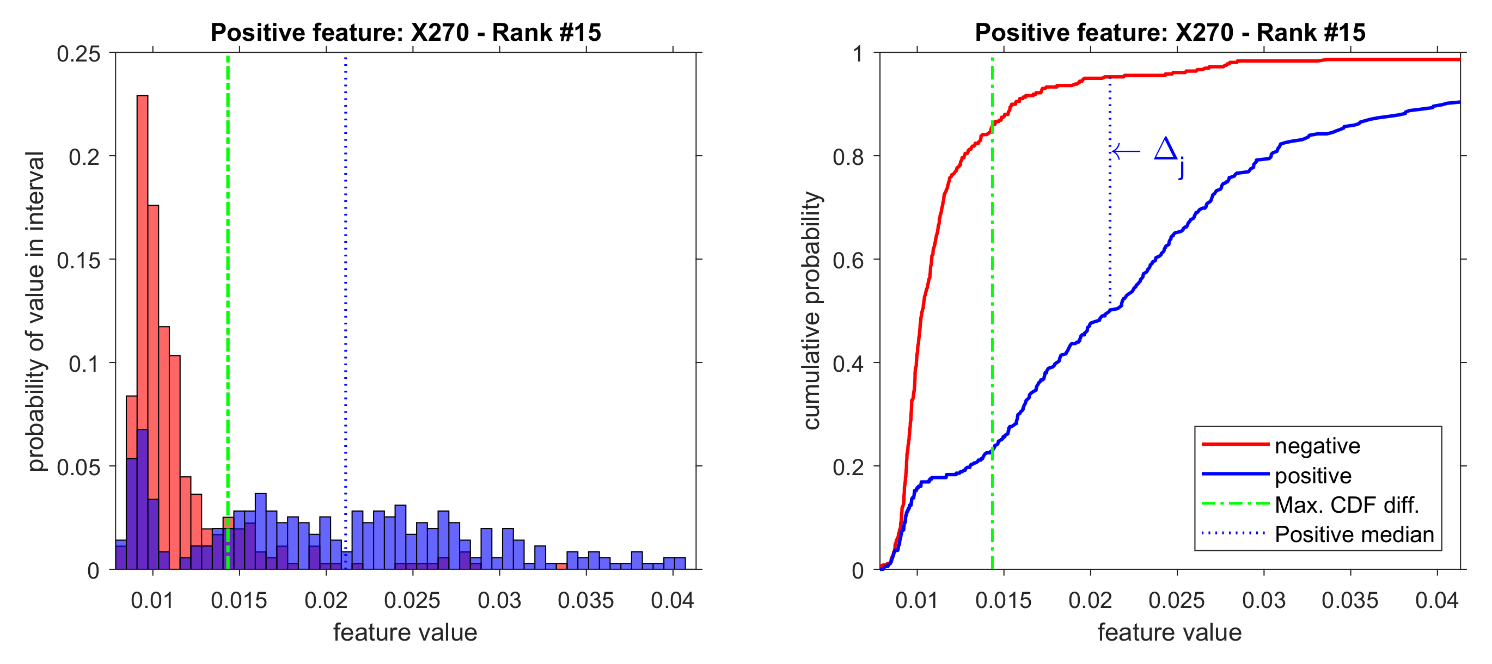

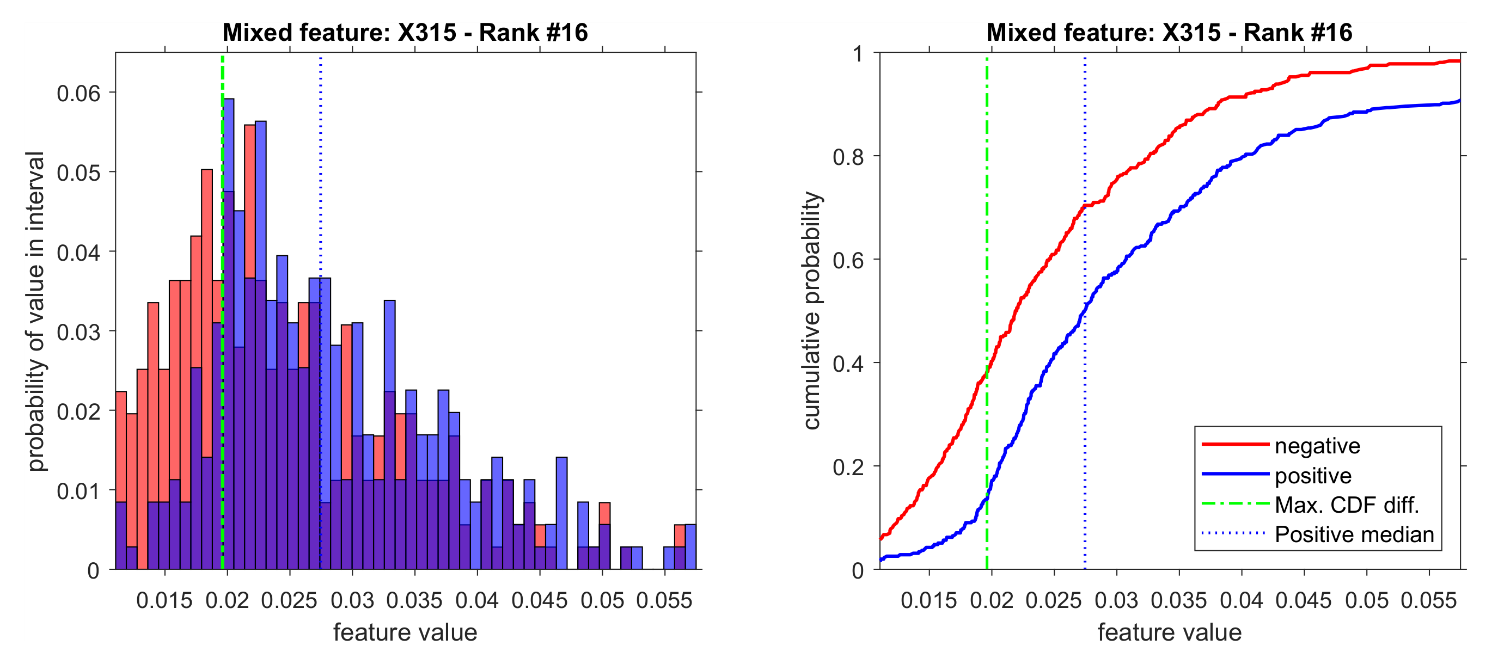

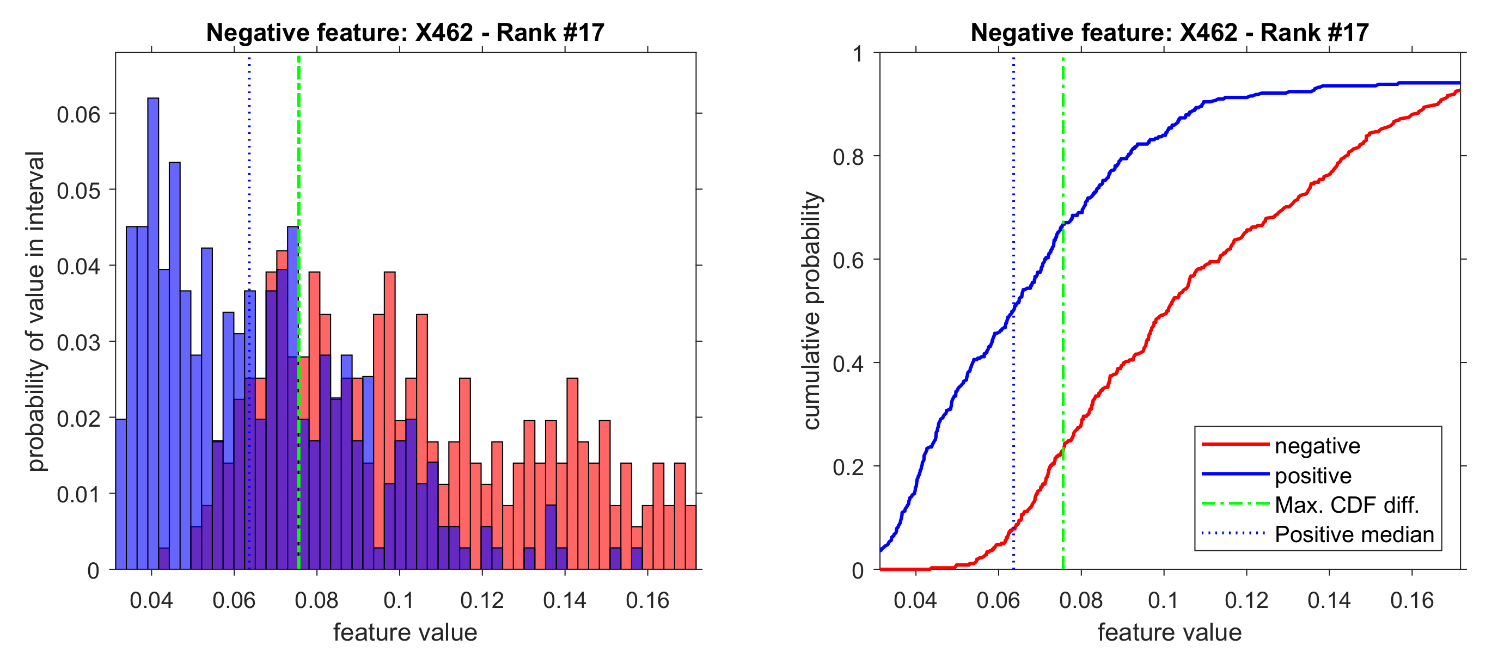

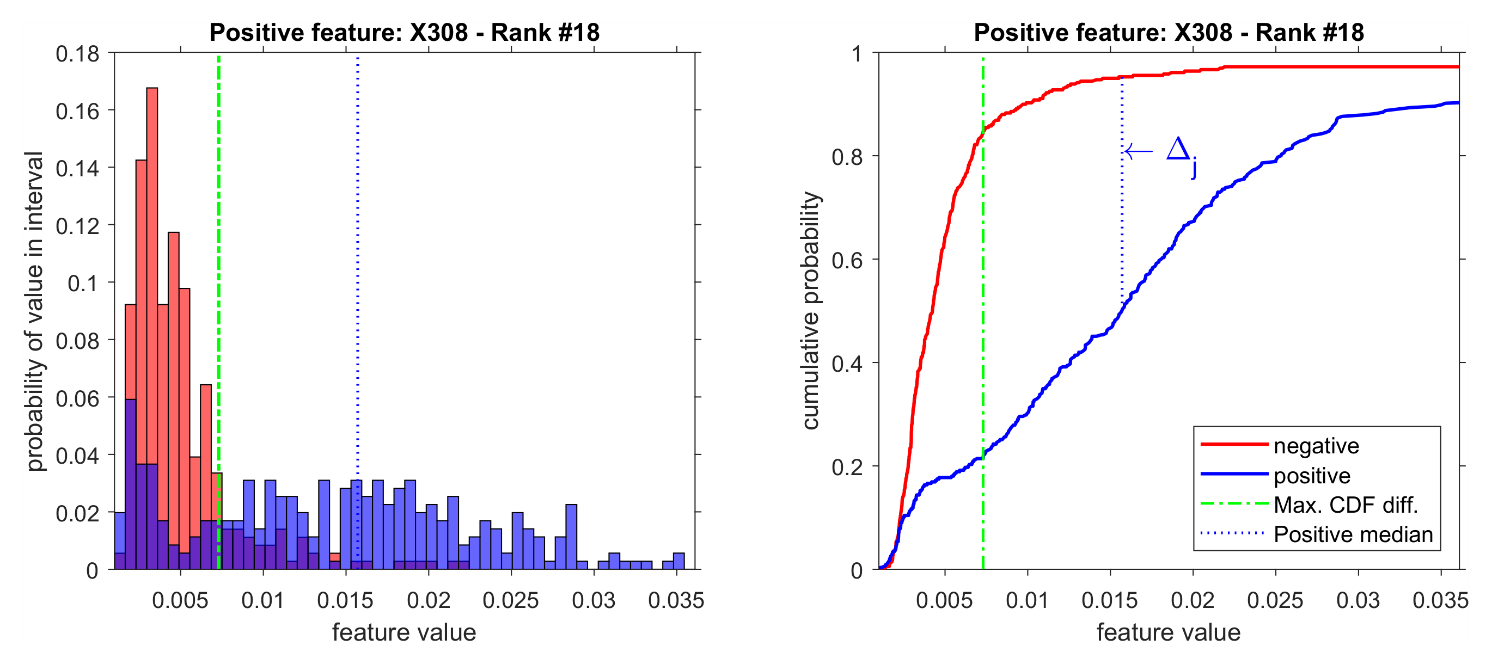


**(c)**

**(b)**

**(e)**

**(d)**

**(f)**

**(h)**

**(i)**

**(g)**

**(k)**

**(j)**

**(l)**

**(m)**

**(n)**

**(o)**

**(p)**

**(q)**

**(r)**

**Figure S1:** Distribution Analysis Chart for 18 Most Discriminant Features considering Probability of value in interval and Cumulative probability. **(a)** Feature 299, **(b)** Feature 673, **(c)** Feature 278, **(d)** Feature 672, **(e)** Feature 263, **(f)** Feature 379, **(g)** Feature 304, **(h)** Feature 657, **(i)** Feature 250, **(j)** Feature 283, **(k)** Feature 389, **(l)** Feature 252, **(m)** Feature 656, **(n)** Feature 395, **(o)** Feature 270, **(p)** Feature 315, **(q)** Feature 462, **(r)** Feature 308.


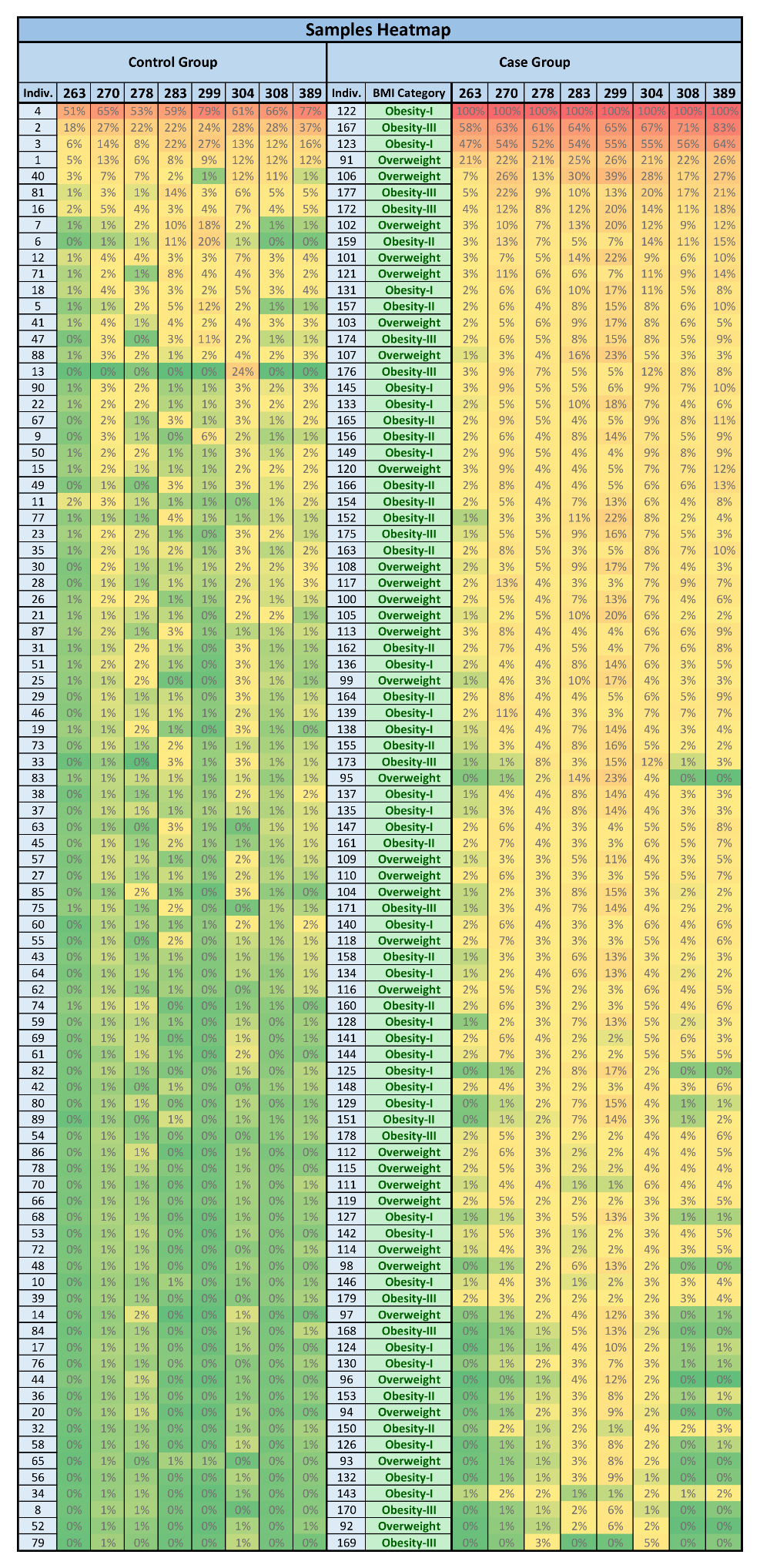


# Figure S2: Heatmap analysis distribution of 5 biomarkers identified through MS and ML process
